# The *Leishmania donovani LDBPK_220120.1* Gene Encodes for an Atypical Dual Specificity Lipid-Like Phosphatase Expressed in Promastigotes and Amastigotes; Substrate Specificity, Intracellular Localizations, and Putative Role(s)

**DOI:** 10.3389/fcimb.2021.591868

**Published:** 2021-03-25

**Authors:** Amalia Papadaki, Olympia Tziouvara, Anastasia Kotopouli, Petrina Koumarianou, Anargyros Doukas, Pablo Rios, Isabelle Tardieux, Maja Köhn, Haralabia Boleti

**Affiliations:** ^1^ Intracellular Parasitism Laboratory, Department of Microbiology, Hellenic Pasteur Institute, Athens, Greece; ^2^ Light Microscopy Unit, Hellenic Pasteur Institute, Athens, Greece; ^3^ Genome Biology Unit, European Molecular Biology Laboratory, Heidelberg, Germany; ^4^ Signalling Research Centres BIOSS and CIBSS, University of Freiburg, Freiburg, Germany; ^5^ Faculty of Biology, University of Freiburg, Freiburg, Germany; ^6^ Team «Biomechanics of Host Parasite Interactions», Institut for Advanced BioSciences, Univ. Grenoble Alpes, Inserm U1209 - CNRS UMR 5309, 38700 La Tronche, France

**Keywords:** *Leishmania* developmental transitions, atypical lipid phosphatase, phosphoinositide signaling and metabolism, P-Tyr/PI phosphatase, flagellar pocket, endocytosis/exocytosis

## Abstract

The intracellular protozoan parasites of the *Leishmania* genus are responsible for Leishmaniases, vector borne diseases with a wide range of clinical manifestations. *Leishmania (L.) donovani* causes visceral leishmaniasis (kala azar), the most severe of these diseases. Along their biological cycle, *Leishmania* parasites undergo distinct developmental transitions including metacyclogenesis and differentiation of metacyclic promastigotes (MPs) to amastigotes. Metacyclogenesis inside the *phlebotomine* sandfly host’s midgut converts the procyclic dividing promastigotes to non-dividing infective MPs eventually injected into the skin of mammalian hosts and phagocytosed by macrophages where the MPs are converted inside modified phagolysosomes to the intracellular amastigotes. These developmental transitions involve dramatic changes in cell size and shape and reformatting of the flagellum requiring thus membrane and cytoskeleton remodeling in which phosphoinositide (PI) signaling and metabolism must play central roles. This study reports on the *LDBPK_220120.1* gene, the *L. donovani* ortholog of *LmjF.22.0250* from *L. major* that encodes a phosphatase from the “Atypical Lipid Phosphatases” (ALPs) enzyme family. We confirmed the expression of the *LDBPK_220120.1* gene product in both *L. donovani* promastigotes and axenic amastigotes and showed that it behaves *in vitro* as a Dual Specificity P-Tyr and monophosphorylated [PI(3)P and PI(4)P] PI phosphatase and therefore named it *Ld*TyrPIP_22 (L*eishmaniad onovani* Tyrosine PI Phosphatase, gene locus at chromosome 22). By immunofluorescence confocal microscopy we localized the *Ld*TyrPIP_22 in several intracellular sites in the cell body of *L. donovani* promastigotes and amastigotes and in the flagellum. A temperature and pH shift from 25°C to 37°C and from pH 7 to 5.5, induced a pronounced recruitment of *Ld*TyrPIP_22 epitopes to the flagellar pocket and a redistribution around the nucleus. These results suggest possible role(s) for this P-Tyr/PI phosphatase in the regulation of processes initiated or upregulated by this temperature/pH shift that contribute to the developmental transition from MPs to amastigotes inside the mammalian host macrophages.

## Introduction

*Leishmania* parasites, a class of trypanosomatid protozoans of the kinetoplastidae family, parasitize both invertebrate (sandflies of the genus *Phlebotomus* or *Lutzomyia*) and vertebrate hosts. When transmitted to the mammal host by the sandfly bite upon blood-feeding, *Leishmania* parasites are responsible for leishmaniases, a wide spectrum of diseases, which are major public health threats in endemic areas (WHO/PAHO, 2020). The most severe and potentially fatal form of these diseases transmitted by *Leishmania (L.) donovani* is visceral leishmaniasis (kala-azar) (Burza et al., 2018; WHO/PAHO, 2020). Up to 1 million new cases of all forms of leishmaniasis occur annually in about 100 endemic countries and over 20,000 deaths are attributed annually to them (WHO/PAHO, 2020). The few anti-leishmania drugs available to date present serious limitations. They are associated with severe side effects/toxicity or teratogenicity, high cost or emergence/spread of drug-resistant parasites in the field (Ghorbani and Farhoudi, 2018). Therefore, there is an urgent need to develop new specific and safe anti-parasitic compounds. In such framework, the identification of molecular determinants along the complex life cycle of these parasites should enlarge the target repertoire for designing anti-parasitic strategies.

To achieve its complex life cycle in the hematophagous insect host and in the mammalian host, *Leishmania* spp. undergo a series of differentiation processes that give rise to distinct biological stages allowing tight adjustments in each of their hosts (e.g., the insect’s luminal digestive tract and the interior of mammalian cells) that are significantly different in temperature, pH, nutrient availability and immune status (Bates, 1994; Mauel, 1996; Sunter and Gull, 2017). The main *Leishmania* developmental forms are: 1) the flagellated promastigotes, living in the sandfly host vector’s digestive tract (Bates, 1994; Dostalova and Volf, 2012) and eventually delivered into the mammalian host skin and 2) the ovioid amastigotes with a short mostly internal flagellum (Glaser et al., 1990; Gupta et al., 2001; Wheeler et al., 2015), propagating within macrophages of the vertebrate hosts inside a modified parasitophorous phagolysosome [(Desjardins and Descoteaux, 1997), reviewed by (Mauel, 1996; Handman and Bullen, 2002; Young and Kima, 2019)]. In the digestive tract of the insect, the dividing promastigotes go through several stages of differentiation the last step of which is called metacyclogenesis (Bates, 1994; Rogers et al., 2002; Bates, 2007). This developmental phase leads to the emergence of infectious metacyclics, short and slender flagellated forms never seen in division. They are highly motile and unattached to the insect’s digestive track and have a flagellum at least twice the length of their cell body (Bates, 2007; Sunter and Gull, 2017). The metacyclic promastigotes are believed to be the only forms injected into the skin when an infected fly takes a blood meal. Metacyclogenesis can be mimicked *in vitro* by cultivating promastigotes under chemically defined conditions allowing the generation of intermediate differentiation forms as well as fully differentiated metacyclic *Leishmania* promastigotes (Bates et al., 1992; Zakai et al., 1998; Nayak et al., 2018). Conditions have also been defined to reproduce the developmental transition from metacyclic promastigotes (MPs) to amastigotes by shifting stationary phase promastigotes’ cultures to 37°C and pH 5.5, mimicking thereby the conditions inside the parasitophorous phagolysosomes of mammalian macrophages (Doyle et al., 1991; Debrabant et al., 2004; Zilberstein, 2020)

The developmental transitions of metacyclogenesis and MP to amastigote enable *Leishmania* parasites to cope with the distinct microenvironments of their hosts by adjusting gene expression (Iantorno et al., 2017; Coutinho-Abreu et al., 2020) and consequently metabolism. These adaptations are reflected by the broad alterations in the absolute and relative RNA and protein levels and activity observed over metacyclogenesis and MP to amastigote transiton [(De Pablos et al., 2016; Inbar et al., 2017) recently reviewed by (Karamysheva et al. (2020)]. In this context, changes in the phosphorylation state of many proteins carried out by specific kinases and phosphatases have already been highlighted (Nascimento et al., 2003; McNicoll et al., 2006; Nascimento et al., 2006; Zhou et al., 2006; Depledge et al., 2009; Morales et al., 2010; Tsigankov et al., 2013; Soulat and Bogdan, 2017) pointing to the importance of P-Tyr phosphatases (PTPs) in the differentiation process of *Leishmania* parasites although few proteins (0.4%) were found to be phosphorylated on tyrosine during the *L. donovani* promastigotes’ differentiation to amastigotes *in vitro* (Tsigankov et al., 2013).

Besides the human homologs of PTPs expressed in *Leishmania* (Nascimento et al., 2006; Soulat and Bogdan, 2017), atypical Dual Specificity Phosphatases (aDSPs) with P-Tyr phosphatase activity have been detected in a phosphatome analysis of *Leishmania* genome sequences (Brenchley et al., 2007). These phosphatases seem to have no human homologs or to be very divergent from human homologs (Brenchley et al., 2007; Soulat and Bogdan, 2017). Moreover, another Bioinformatics study identified four *Leishmania* aDSPs as members of an “Atypical Lipid Phosphatase” family (ALPs) (Beresford et al., 2010), a group of enzymes found only in bacteria and lower eukaryotes. All ALPs share the characteristic structural P-loop feature (HCXXGKDR) of the catalytic site of PTPs, a signature also found in MptpB, a triple specificity (i.e., P-Tyr, P-Ser/P-Thr, and PI) phosphatase from *M. tuberculosis* (Beresford et al., 2007) and the LipA, a P-Tyr, and PI phosphatase from *Listeria monocytogens* (Kastner et al., 2011). Of note, both MptpB and LipA are secreted by the bacteria into their respective host cells where they subvert PI signaling, hence contributing to bacteria virulence (Beresford et al., 2007; Kastner et al., 2011). One of the *Leishmania* members of the ALP family encoded by the *Lmj*F.22.0250 gene was shown in the same study to dephosphorylate P-Tyr peptides and monophosphorylated PIs. Interestingly, the *Lmj*F.22.0250 gene transcript was later reported to be highly enriched during metacyclogenesis in the natural sandfly vector (Inbar et al., 2017). In the same line, *L. mexicana* parasites in which was ablated the *LmxM*.22.0250 gene, orthologous to the *Lmj*F.22.0250, showed severely impaired survival in primary mouse macrophages in an *in vitro* infection system (Kraeva et al., 2019), results supporting a strong contribution of this ALP to the parasite’s fitness. Overall, the unique sequence- and biochemical- related features of ALPs, in addition to their acting as fitness determinants, make them attractive candidate targets for the development of specific antimicrobial inhibitors/drugs.

Herein, we report data from the biochemical characterization of the *Ld*TyrPIP_22 protein (***L*** *eishmania* ***d*** *onovani* **Tyr**osine **PI P**hosphatase with gene locus at chromosome **22**), encoded by the *LdBPK_220120* gene, the *L. donovani* ortholog of the *LmjF.22.0250* and *LmxM.22.0250* genes mentioned above. We found that the *Ld*TyrPIP_22 sequence is highly conserved in all sequenced *Leishmania* spp. from the *Leishmania* and *Sauroleishmania* subgenera and detected the expression of the *LdBPK_220120* encoded protein in different morphological forms of cultured *L. donovani* promastigotes and axenic amastigotes. *Ld*TyrPIP_22, when expressed in bacteria, dephosphorylates P-Tyr peptides and monophosphorylated PIs similarly to its *L. major* ALP ortholog. It is specifically distributed in distinct subcellular compatments in the promastigote and amastigote cells and is recruited to the flagellar pocket upon a temperature shift from 25°C to 37°C, one of the main parameters that drive the developmental transition of the *Leishmania* MP to amastigote.

## Materials and Methods

### Reagents and Antibodies

All chemicals used, unless otherwise stated, were of analytical grade and purchased from Sigma-Aldrich or Applichem. Specifically, Digitonin (D141-100MG), Triton-X 100 (X100-100ML) and Ponceau S (P3504-50G) were from Sigma-Aldrich. Nourseothricin (NTC-AB-102L0) was from Jena Biosciences. Restriction enzymes were purchased from Roche (New England Biolabs) and/or KAPA Biosystems. Taq DNA polymerase (R001A) and T4 ligase (2011B) were from TaKaRa. All primers used in the PCR reactions (synthesized by VBC Biotech) are listed in **Table S1**. DNase I (2270A) was from TaKaRa and RNAse (10109134001) from Sigma Aldrich (Merck). One kb DNA ladder (N3232L) was from New England Biolabs (NEB), protein molecular mass standards (17-0446-01) were purchased from Amersham Biosciences and Nippon Genetics (Broad range:10–180 kDa, MWP03) while protein quantification reagent Bradford (B6916) and proteolytic inhibitors (P8465) were from Sigma-Aldrich. Fetal Bovine Serum (FBS) was from Thermo Fisher Scientific (10270106, Gibco) or Biosera (FB-1001/500). Bacto-tryptone (211705), Bacto Yeast extract (212750), and Bacto-agar (14050) were from BD Biosciences. The mouse monoclonal 6xHistidine epitope tag antibody (Ab) was from Acris Antibodies (SM1693PS), the a-tubulin (T5168) mouse monoclonal (mAb) was from Sigma, the rabbit polyclonal (pAb) a-Leish actin was kind gift from Dr Amogh Sahasrabuddhe (Division of Molecular and Structural Biology Central Drug Research Institute Lucknow, India). The GAPDH pAb was a kind gift of Frédéric Bringaud (U. of Bordeaux/CNRS, France). The a-mRFP pAb was prepared by us as described by Papadaki et al. (2015). The a-A2 Ab was from Abcam (ab150344). The a-EF1a Ab clone CBP-KK1 (05-235) was purchased from Merck. All Fluorochrome-conjugated secondary Abs [Alexa Fluor^®^ 546 (A-11030 or A-11035) and Alexa Fluor^®^ 488 (A32723 or A32731)] were from Thermo Fisher Scientific. Goat anti-rabbit HRP (41460) and goat anti-mouse HRP (31230) were from Pierce while the a-mouse and a-rabbit Abs conjugated with CF488A were from Biotinum (20010 and 20012, respectively). Hoechst 33342 (H3570) and FM 4-64 FX fixable membrane stain (F34653) were purchased from Thermo Fisher Scientific.

### Cell Culture

The murine monocytic cell line J774 was cultured in high glucose RPMI (1640) (Biosera LM1638) containing 10% (v/v) hiFBS [heat-inactivated (56°C, 30 min) fetal bovine serum], 1 U/ml penicillin and 0.1 mg/ml streptomycin. Cell counting was performed with a Neubauer hemocytometer.

*L. donovani* (strain LG13, MHOM/ET/0000/HUSSEN) promastigotes were cultured in RPMI 1640 containing 10% (v/v) hiFBS 1 U/ml penicillin, 0.1 mg/ml streptomycin (Gibco) and 10 mM Hepes (Gibco), at 25°C as previously described (Papadaki and Boleti, 2019). Cell counting was performed with a Malassez hemocytometer as described (Papadaki and Boleti, 2019). *L. donovani* axenic amastigotes were obtained according to modified published protocol (Barak et al., 2005; Zilberstein, 2020). Briefly, promastigotes at the stationary phase of growth, obtained after 8 days incubation at 25°C, pH 7, were harvested and resuspended in prewarmed (37°C) Medium 199 (pH of 5.5) containing 25% v/v FBS. Axenic amastigotes were obtained after 120 h incubation under these conditions (37°C, pH 5.5). Parasites incubated in the conditions inducing transformation of promastigotes to amastigotes (37°C, pH 5.5) were also analyzed after 24 h.

### DNA Constructs and Cell Transfection

The gene encoding the *Ld*TyrPIP_22 [1-258 amino acids (aa), GenBank^®^, accession No MF461274] was amplified by PCR from genomic *L. donovani* DNA (strain LG13) and inserted into the *BglII* site of the pLexsy-sat-*mrfp1* plasmid (Papadaki et al., 2015) to create the pLexsy-*ld*t*yrpip__2_*_2_-*mrfp1* plasmid. Secondly, the *ldtyrpip_*_22_ gene was cloned into the *BglII*/*XhoI* sites of the pTriEx1.1 vector (Invitrogen), in frame with the C-terminal His tag to produce the pTriEx1.1-*ldtyrpip_*_22_ and pTriEx1.1-N15-*ldtyrpip_*_22_ plasmids. N15 corresponds to the 15 additional aa (Met-Ala-Ile-Ser-Arg-Glu-Leu-Val-Asp-Pro-Asn-Ser-Gln-Ile-Ser) added to the N-terminus of *Ld*TyrPIP_22 by expression from the second construct. All plasmid constructs were propagated in the *Escherichia coli* (*E. coli*) Top10F’strain for small (mini) and large scale (midi) plasmid DNA preparations. Two positive clones were selected and sequenced in each case (VBC-Biotech). The results showed 100% nucleotide sequence identity at the DNA level between the two clones. For recombinant protein production we used clones of *E. coli* BL21 (DE3) strain harboring the pTriEx1.1 based plasmids.

The transgenic promastigotes were generated based on minor modifications of a protocol previously described (Papadaki et al., 2015). Briefly, for episomal expression of *Ld*TyrPIP_22-mRFP1, *Leishmania* promastigotes (2x10^7^ cells/ml) at the stationary phase of growth were transfected by electroporation with supercoiled circular pLexsy-sat-*ldtyrpip_*_22_ *-mrfp1* plasmid. For electroporation, *Leishmania* promastigotes from a 10 ml culture were washed once and incubated (10 min, on ice) in 10 ml ice cold electroporation buffer (21 mM HEPES, pH 7.5, 0.7 mM Na_2_HPO_4_, 137 mM NaCl, 6 mM glucose, 5 mM KCl). Subsequently, they were centrifuged (1,000 *g*, 10 min), resuspended in 1 ml ice cold electroporation buffer and 400 *u*l of this suspension (2 x10^8^ cells/ml) were added in a 1.5 ml Eppendorf tube containing 20-50 *μ*g plasmid DNA dissolved in 50 *μ*l dH_2_O. After mixing, the promastigotes’/DNA suspension was transferred to chilled electroporation cuvette (Gene Pulser/Micro Pulser Electroporation cuvette 0.2 cm, 165-2086, BioRad) and electroporated (50 *μ*F, 0.45 kV, pulse time ~ 4-5 msec). The electroporated promastigotes (8x10^7^ cells), after a 10 min incubation on ice, were transferred in 10 mL RPMI [20% (v/v) hiFBS] and incubated for 16 h at 25°C before the addition of the antibiotic Nourcethricin. A pool of positive transgenic promastigotes was selected by the addition (once a week) of gradually increasing concentration (20*–*100 *μ*g/ml) of Nourcethricin.

### Overexpression and Purification of Recombinant *Ld*TyrPIP_22-His Protein

The recombinant proteins used in the biochemical characterization of *Ld*TyrPIP_22 were produced in *E. coli* as follows: A 4 L culture of BL21 (DE3) cells carrying the plasmid pTriEx1.1-rN15-*ldtyrpip_*_22_-His was induced overnight (~16 h) with 0.5 mM isopropyl *β*-D-thiogalactoside (IPTG) at 20°C and the cells were harvested by centrifugation (4,000 *g*, 15 min) 20 h later. Subsequently, the cell pellet was resuspended in lysis buffer (50 mM Tris, 300 mM NaCl, 30 mM imidazole, pH 8) with proteolytic inhibitors (Sigma, P 2714) and lysed by a freeze-thaw process repeated three times followed by 6*–*8 sonications (30*–*60 s, 100 W) each followed by a 30 s pause step of incubation in ice. The fusion rN15-*Ld*TyrPIP_22-His protein was purified by nickel-affinity chromatography. The soluble fraction of the bacterial lysate was passed through a GE Healthcare (His TrapTM HP) His-binding column (ÄKTA-FPLC) with Ni^2+^ charged resin. The bound proteins, after been washed with lysis buffer, were eluted with 50 mM Tris, 300 mM NaCl (pH 8) and gradient concentration of imidazole up to 400 mM. As most of the proteins were eluted at fraction 1 (200 mM imidazole), this was used for a second purification step through a Talon column (Clontech Laboratories/A TaKaRa Bio company) packed with Co^2+^ charged resin. Finally, a total amount of ~1.6 mg of highly purified protein was obtained.

### Generation of Antibodies

The fusion protein rN15-*Ld*TyrPIP_22-His purified by Metal-Affinity Chromatography (Qiagen Ni–NTA Superflow resin) was injected into New Zealand white rabbits and BALB/c mice to raise polyclonal anti-sera, according to published protocols (Papadaki et al., 2015). All experimental procedures were approved by the Institutional Animal Bioethics Committee following the EU Directive 2010/63 and the National Law 2013/56. Purified rabbit anti-*Ld*TyrPIP_22 pAb was obtained by low pH elution from immunoblots of purified rN15- *Ld*TyrPIP_22-His, as previously described (Harlow and Lane, 1988).

### Phosphatase Activity Assays

Initially, the rN15-*Ld*TyrPIP_22-His phosphatase activity was assayed at 25°C and 37°C in a reaction buffer (100 mM Tris, 150 mM NaCl, 4mM DTT) containing 10 mM of the generic phosphatase substrate p-nitrophenyl phosphate (*p*NPP) and pH ranging from 4 to 8. The reaction (200 *μ*l buffer with substrate) was initiated by the addition of enzyme and quenched after 60 min by addition of two volumes 0.5 N NaOH. The absorbance of the reaction product (*p*-nitrophenolate = *p*NP) in the supernatant was measured at λ=405 nm.

To study the kinetic parameters of rN15-*Ld*TyrPIP_22-His, phosphatase assays were carried out three times in triplicates, in a total volume of 60 *μ*l, containing reaction buffer (pH 6), enzyme (2 *μ*M), and *p*NPP (0.5–100 mM) at 25°C. The absorbance (λ=405 nm) was measured in Infinite M1000PRO plate reader (TECAN). Kinetic constants were determined by fitting the data to the Michaelis-Menten equation using GraphPad Prism Software 5.01 (GraphPad, San Diego, CA).

### Substrate Specificity and Inhibition Assays

To determine the substrate specificity of rN15-*Ld*TyrPIP_22-His a number of reactions were carried out using as substrates P-Tyr (1 mM) or P-Ser/P-Thr (60 *μ*M) peptides in reaction buffer (100 mM Tris, 150 mM NaCl, 4 mM DTT, pH 7) at 25°C (**Table S2**). The peptides were synthesized and purified according to standard procedures. Additionally, mono, bis, or trisphosphorylated PIs (100 *μ*M) (Avanti polar lipids or Echelon) (**Table S2**) were tested as plausible substrates of the recombinant *Ld*TyrPIP_22 using the EnzChek^®^ (Thermo Scientific) protocol (pH 7, 25°C) following the manufactures’ instructions. The absorbance reading at 360 nm was performed with the X Infinite M1000PRO plate reader (TECAN).

The effect of inhibitors was evaluated in phosphatase assays where rN15-*Ld*TyrPIP_22-His solutions (2 *μ*M) were pre-incubated (25 min, RT) with a) sodium orthovanadate (Na_3_VO_4_) concentrations ranging from 1*–*20 mM, b) 100 mM sodium fluoride (NaF) or c) with reaction buffer only (100 mM Tris, 150 mM NaCl, 4 mM DTT, pH 7). The phosphatase activities in the solutions were subsequently assayed using as substrate the *p*NPP (10 mM) and reading the absorbance at λ=405 nm (max absorbance of the chromogenic product *p*NP). Estimation of the product concentration was according to a standard curve generated with *p*NP samples of known concentration.

### Preparation of Total Lysates from *Leishmania* Promastigotes

Promastigotes were resuspended in lysis buffer [20 mM Tris-HCl (pH 6.8), 0.1% SDS] (10^8^ parasites in 100 µl lysis buffer), boiled for 5 min, cooled on ice and treated with DNase I (5*–*10 U/sample, 15 min, on ice) to remove DNA. Protein was estimated by the Bradford assay method. For further analysis by SDS-PAGE, 6X Laemmli sample buffer was added and the samples were either boiled for 5 min or incubated at 37°C for 30 min in the presence of proteolytic inhibitors.

### Detergent-Based Protein Fractionation

Digitonin permeabilization of *L. donovani*-r*Ld*TyrPIP_22-mRFP1 stationary phase *L. donovani* promastigotes is based on protocols previously described (Foucher et al., 2006; Doukas et al., 2019) with slight modifications. Briefly, *L. donovani* (*wt* or transgenic) promastigotes (~2 x10^9^) were harvested by centrifugation (1,000 *g*, 7 min, 4°C), washed twice in resuspension buffer (145 mM NaCl, 11 mM KCl, 75 mM Tris-HCl, pH 7.4) resuspended in 0.5 ml of the same buffer and supplemented with protease inhibitors. A stock of 100 mM digitonin solution in dH_2_O was prepared. From this 100 mM digitonin solution were subsequently prepared by serial dilution solutions of 20 mM, 2 mM, 400 *μ*M and 40 *μ*M. Membrane permeabilization and protein fractionation were achieved by adding 0.5 ml digitonin solution prewarmed at 37°C of progressively increased detergent concentrations (stepwise, four steps) to achieve digitonin concentrations of 20 *μ*M, 200 *μ*M, 1 mM, or 10 mM. In each step a soluble fraction and a corresponding pellet were recovered. The final pellet recovered after treatment with 10 mM digitonin (F5; enriched in plasma membrane, nuclei and cytoskeletal proteins), was further solubilized with 0.5 ml 1% (v/v) TritonX-100 (1 h, 4°C) and the soluble fraction (F5 S) was recovered from the insoluble fraction (F5 P) by centrifugation (20,000 *g*, 20 min, 4°C). The soluble fractions (F1-F4 and F5 S) were subjected to acetone precipitation by the addition of an acetone (prechilled at -20°C) volume equal to four sample volumes followed by 1h incubation at -20°C. The protein pellets recovered and the F5 P pellet were solubilized in Laemmli buffer, samples were boiled (5 min, 95°C) and further analyzed by SDS-PAGE and Western blot.

### Protein Electrophoresis and Western Blotting

Proteins were separated by SDS-PAGE (12% gel) and transferred to Hybond-C nitrocellulose (Amersham) membrane using a wet blotting apparatus (BioRad). After protein transfer, Hybond-C membranes were stained with Ponceau S solution [0.5% (w/v) Ponceau S dissolved in 1% (v/v) acetic acid]. Nonspecific sites for Ab binding on the nitrocellulose membrane were blocked by incubation (1 h, RT) with Blocking buffer [Tris-buffered saline (TBS; 50 mM Tris-Cl, pH 7.5, 150 mM NaCl), 0.05% (v/v) Tween 20, 5% w/v BSA]. Incubation with the primary Abs was performed overnight (~16 h) at 4°C. The a-*Ld*TyrPIP_22 mouse or rabbit pAbs were used at 1:1200 (serum) or ~1 μg/ml (affinity purified) respectively diluted in [Tris-buffered saline, 0.1% (v/v) Tween 20 (TBST)]. Other Abs were used as indicated in figure legends. After three washes in TBST, the blots were incubated (1 h, RT) with HRP-labeled a-mouse or a-rabbit Abs used at 1:5,000 dilution. Following three washes in TBST and one final wash in TBS Ab reactivity was revealed either by the ECL plus system (Amersham) or by the chromogenic DAB method. In the former case, membranes were exposed to Kodak photographic films further developed with Kodak reagents. Reprobing with the a-mRFP1 pAb (0.5 *μ*g/ml) or a-EF1A (1:10,000) where required, was performed after stripping the membrane by incubation (30 min, RT) with a low pH buffer (25 mM glycine-HCl, 1% (w/v) SDS, pH 2) followed by washing and reblocking with the Blocking buffer. Apparent molecular weights of proteins detected with the specific Abs were assigned by using low (14*–*98 kDa, Amersham) or broad range molecular weight markers (10*–*180 kDa, Nippon).

### Immunofluorescence Staining of *Leishmania* and Mammalian Cells and Confocal Imaging Analysis

*L. donovani* promastigotes were fixed with 2% (w/v) paraformaldehyde (PFA) (20 min, RT) in PBS, allowed to adhere to poly-L-lysine coated coverslips and treated with 50 mM NH_4_Cl in PBS (10 min, RT) followed by PBS wash. Fixed cells were incubated (1 h, RT) first with primary Abs in blocking buffer (PBS, 1% (w/v) BSA) and after extensive washing, the appropriate secondary Abs conjugated to Alexa Fluor^®^ 546 and Alexa Fluor^®^ 488 were added at a final concentration of 2 *μ*g/ml in blocking buffer (1 h, RT). The secondary Abs were removed with extensive washing and the parasite DNA was stained (10 min, RT) either with 10 *μ*g/ml propidium iodide solution in PBS containing 100 *μ*g/ml RNase or with Hoechst 33342 (Mol. Probes H3570) at final concentration of 10 *μ*g/ml. Coverslips were mounted with Mowiol 4-88 (10% (w/v) Mowiol-Calbiochem, 25% (v/v) glycerol, 100 mM Tris-HCl, pH 8.5) on microscope slides, sealed with nail polish, and stored at 4°C.

For the infection of macrophages, stationary phase *L. donovani* promastigotes, obtained after 8 days in culture without subculturing, were added to semi confluent cultures of J774 mouse macrophage cells grown on coverslips in multiwell dishes, at 20:1 parasite/macrophage ratio and the macrophages/parasites co culture were incubated at 37°C for 4 h. At the end of this period, the parasites were removed, the macrophages were washed once with prewarmed (37°C) fresh medium and were either processed for immunofluorescence confocal microscopy analysis or they were further incubated in fresh medium (RPMI, 10% v/v FBS, 1% w/v penicillin/streptomycin, 37°C) according to each experiment’s specification. For immunofluorescence analysis, infected J744 macrophages were fixed (20 min, RT) with PFA [4% (w/v) in PBS]. The non-reacted PFA was neutralized with 50 mM NH_4_Cl in PBS (10 min, RT) and the cells were stained with primary and secondary Abs or phalloidin-Alexa-546^®^. Coverslips were mounted as described in the previous paragraph.

Microscopic analysis of the *Leishmania* cells or the infected J744 macrophages was performed with the Leica TCS SP or SP8 confocal microscopes using the 63X apochromat lens. Image acquisition included collecting z stacks of 0.3 or 1 μm step size for parasites or infected macrophages respectively.

The extent of co localization between the *Ld*Actin and the *Ld*TyrPIP_22 (green and red FL respectively) was measured using the 3D “Coloc” module of Imaris v9.2.1, which utilizes the algorithms introduced by Costes et al. for the automatic selection of thresholds of the image channels (Costes et al., 2004). We have used a third channel as a masking area for the entire analysis (created with the channel Arithmetic function “sqrt (ch1*ch2)”, to exclude the background pixels of the dataset from the co localization analysis. The mask channel is used in conjunction with the automatic threshold function. This way Imaris “3D Coloc” can generate a new channel (the co localization channel), which only contains voxels representing the co localization between the red and green channels. Co localization was quantitated separately in the promastigotes’ cell bodies and the flagella. Threshold 5 was used for analysis in the cell body and 2.9 for the entire promastigote and the flagellum.

### Secretion Assay

*L. donovani* promastigotes in a stationary phase culture (50 ml) were enumerated with a Malassez hemocytometer or by measuring turbidity at OD_600_ (Papadaki and Boleti, 2019) and were then harvested by centrifugation (1,000 *g*, 10 min, RT). The *Leishmania* cell pellet was resuspended for washing in the same volume of RPMI and the cells were collected by centrifugation (1,000 *g*, 10 min, RT). Subsequently the promastigotes’ pellet was resuspended in RPMI/10 mM Hepes (1/5^th^ of the starter’s culture volume) without FBS and the promastigotes were incubated at 25°C for 9 h or at 37°C for 6 h. At the end of the incubation period, the cells were separated from the culture supernatant by centrifugation, washed with PBS and stored (-20°C) until use. The culture supernatant was centrifuged (21,000 *g*, 20 min, 4°C) and filtered through a 0.2 *μ*m filter to remove cell debris. Finally, the proteins in the culture supernatant were precipitated by adding 4 volumes of prechilled acetone (-20°C), vortexing and incubating overnight at -20°C. The precipitated proteins were collected by centrifugation at 14,000 g and recovered by decanting the supernatant. Acetone was allowed to evaporate from the uncapped tube at room temperature for 30 min. Appropriate volume of buffer for the downstream process was added and the sample was vortexed thoroughly to dissolve the protein pellet. Alternatively, the culture supernatant was concentrated through 10 kDa cutoff centrifugal filters (Merck) and the buffer was changed to 20 mM Tris-HCl, 150 mM NaCl, pH 9. The same buffer was used to equilibrate a Superdex 200 10/300 column which was subsequently used for analysis of the supernatant proteins according to molecular size. After fractionation, the samples were concentrated with the use of filters (10 kDa MW cutoff, Thermo Fischer) and stored at -20°C until analyzed by SDS-PAGE and WB.

### Bioinformatics and Statistical Analysis

The algorithms used for identifying sequence similarities prediction (Blast) and multiple sequence alignment/editing were: 1) TriTrypDB BLAST ^1^ 2) ClustalW2^2^; 3) BioEdit Sequence Alignment Editor, version 7.0.9.0 (Ibis Biosciences). Signal P 3.0^3^) and NetPhos3.1b; 4) ExPaSy ProScale Hphob./Kyte & Doolittle^4^; 5) NetPhos3.1b tool^5^ and GPS-Lipid^6^ were algorithms used to predict sites for post-translational modifications, signal sequence for secretion. The sequence used in this study to design primers for PCR amplifications of the *ldtyrpip_*_22_ gene from *L. donovani* is the ortholog *LINF_220007400* from *L*. *infantum* [(clone JPCM5 (MCAN/ES/98/LLM-877)] (TritrypDB) (**Table S1**). To perform the Blast search for *ldtyr*pip_22 orthologs in *Leishmania* spp. genome, we used the *ldtyrpip_*_22_ sequence registered in GenBank as ‘*Leishmania donovani* tyrosine phosphoinositide phosphatase, *Ld*TyrPIP_22’ (*accession No* MF461274). The rooted phylogenetic tree analysis was based on the UPGMA method. Data for the Enzyme kinetics were analyzed using the GraphPad Prism Software 5.01 Michaelis-Menten model algorithm^7^. Graphs of enzyme activities were generated with the use of GraphPad. Standard deviations and t-test for statistical significance were calculated using the Excel’s Formula and Data tools. The Icy computational algorithm^7^ ^,^ ^8^ and the Adobe Photoshop CS6 Portable were used for image evaluation and processing, while for the co localization analysis, we used the 3D “Coloc” module of Imaris v9.2.1. The Fiji algorithm was used for quantification of the band intensities in the Western Blot digital images.

## Results

### The *LDBPK_220120.1* Gene Product *Ld*TyrPIP_22 Is Highly Conserved Among the *Leishmania* and *Sauroleismania* Subgenera of *Leishmania* spp. and Shows High Homology to Pathogenic Bacteria Virulence Factors

**Figure 1:**
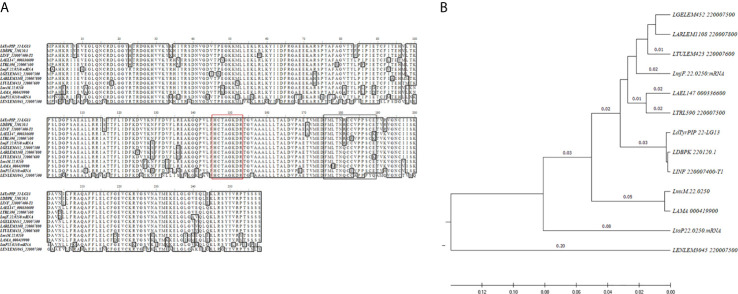
Sequence comparison of the *Ld*TyrPIP_22 orthologs in *Leishmania* spp. **(A)** ClustalW multiple sequence alignment of the *ldtyrpip__22_* gene product orthologs in *Leishmania* spp. The *L. donovani* (strain LG13, MHOM/ET/0000/HUSSEN) *Ld*TyrPIP_22 aa sequence (GenBank^®^ accession No MF461274) was generated in this work. The aa sequences for the *L. donovani LdBPK_220120.1*gene product from the Nepalese strain BPK282A1 and from another eleven *Leishmania* spp. (**Table S2**) with genomes sequenced were obtained from the TriTrypDB database and were analyzed/edited using the software BioEdit Sequence Alignment Editor. The aa of the PTP P-loop motif are framed by a red box; The aa predicted as S-Palmitoylation site are framed by the black box. **(B)** Rooted phylogenetic tree (UPGMA) from a multiple sequence alignment of the *Ld*TyrPIP_22 orthologs from *Leishmania* spp.

Our interest on PI phosphatases in *Leishmania* spp. focused on a study identifying the *Leishmania major LmjF.22.0250* gene that encodes for a dual specificity PI and P-Tyr phosphatase (Beresford et al., 2010). This protein (LM1) contains the putative P-loop motif (HCXXGKDR[TA]G) of PTPs detected in the Mptpb triple specificity PI phosphatase from *M. tuberculosis* and in the LipA dual specificity P-Tyr and PI phosphatase from *L. monocytogenes* (Beresford et al., 2010). Moreover, a later study showing an increase in the levels of the *LmjF.22.0250* gene transcripts during metacyclogenesis in the natural sandfly vector (Inbar et al., 2017) prompted us to better characterize the structural and functional features of this phosphatase in the related *L. donovani* species, which causes in humans the potentially fatal disease of visceral leishmaniasis (Burza et al., 2018). We therefore cloned the *LmjF.22.0250* ortholog gene from *L. donovani* by a PCR approach using genomic DNA isolated from the *L. donovani* LG13 strain (MHOM/ET/0000/HUSSEN) (*Materials and Methods*; Gene Bank accession No MF461274). This was found to be 99.9% identical to the *LDBPK_220120.1* gene (777 bp) from the sequenced *L. donovani* Nepalese strain BPK282A1 registered in the TriTrypDB database with one base difference leading to substitution of Ala^171^ by a Thr^171^ (**Figure 1A**). The *LDBPK_220120.1* gene, from now on designated in our study as *ldtyrpip__22_ (L eishmania d onovani* Tyrosine PI Phosphatase on chromosome 22*)*, is located at positions 91159 to 91935 of chromosome 22. A Blast search using the full length *ldtyrpip__22_*gene product from the *L. donovani*, strain LG13, identified orthologs in all *Leishmania* spp. genome sequences available in the TriTrypDB with exception the *L. braziliensis* strain M2904, MHOM/BR/75M2904 from the *Viania* subgenus. In this case a pseudogene (*LbrM.22.2.000240*) was retrieved by our search. Multiple sequence alignment indicated that the *Ld*TyrPIP_22 protein sequence (length 258 aa, calculated MW: 29081.69 Da, PI: 8.30) is highly conserved (81.34–99.22% identity) (**Figure 1**, **Table S2**) in several species of the *Leishmania* and *Sauroleismania* subgenera of the *Leishmania* genus according to a recent classification of *Leishmania* spp. (Akhoundi et al., 2016; Akhoundi et al., 2017). The rooted phylogenetic tree (UPGMA) from a multiple sequence alignment of the *Ld*TyrPIP_22 orthologs from several *Leishmania* spp. (**Figure 1B**) highlighted the sequence similarities depicted in the representation of the ClustalW2 sequence analysis shown in **Figure 1A** and **Table S2**. Interestingly, no homologs were detected by this BLAST analysis in the closely related species of *Trypanosoma*.

With respect to its bacteria homologs (Beresford et al., 2010) from *M. tuberculosis* (MptpB, GenBank: CCC62750.1), *L. monocytogenes* [lmo1800, NP_465325; lmo1935, NP_465459)], *Yersinia pestis* KIM10+ (WP_002228183), and *Bacillus anthracis* strain Ames (NP_845680), the *Ld*TyrPIP_22 shows in its entire sequence length 19.4%, 29.6%, 36.1%, 27.4% and 32.4% aa sequence identity, respectively. In the P-loop active site-containing region the sequence identity is 73-91% with the respective protein sequences of the aforementioned bacterial homologs (**Figure S1**).

Finally, by applying several tools of bioinformatics, we searched for predicted structural motifs and post-translational modifications in the *Ld*TyrPIP_22 aa sequence. As the bacteria homologs of this putative phosphatase are secreted, we first analyzed by the SignalP 3.0 software for the presence of a eukaryotic secretion signal sequence motif in the N-terminus of *Ld*TyrPIP_22. No such sequence was detected. However the absence of a secretion signal does not exclude the possibility that *Ld*TyrPIP_22 is secreted since a significant number of *Leishmania* proteins with no signal sequence motif are secreted *via* exosomes (Silverman et al., 2008; Dong et al., 2019) or ectosomes [recently reviewed by (de Souza and Barrias, 2020)]. Subsequently, we searched for putative phosphorylation sites using the NetPhos3.1b bioinformatics tool. Interestingly, the extreme C-terminal Ser residue of a series of Ser and Thr residues (TSSSS) was predicted to be phosphorylated with high score (0.970). Three more residues in the N-terminal half of the molecule, Ser^74^, Ser^116^, and Tyr^127^, were also predicted to be phosphorylated (scores 0.991, 0.973, and 0.945, respectively). This information, suggests that the *Ld*TyrPIP_22 may be post-translationally regulated by phosphorylation. Moreover, the *Ld*TyrPIP_22 sequence was checked for other possible post translational modifications. In this search, a putative palmitoylation site at Cys^182^ (S-Palmitoylation: Cluster C: FMLTNR**C**CVPPSCE) in the *Ld*TyrPIP_22 sequence was revealed by the GPS-Lipid algorithm with moderately high score (1.495 with cutoff 1.396).

### Recombinant Bacterially Expressed r*Ld*TyrPIP_22-His Acts as a PI and P-Tyr Dual Specificity Phosphatase

**Figure 2:**
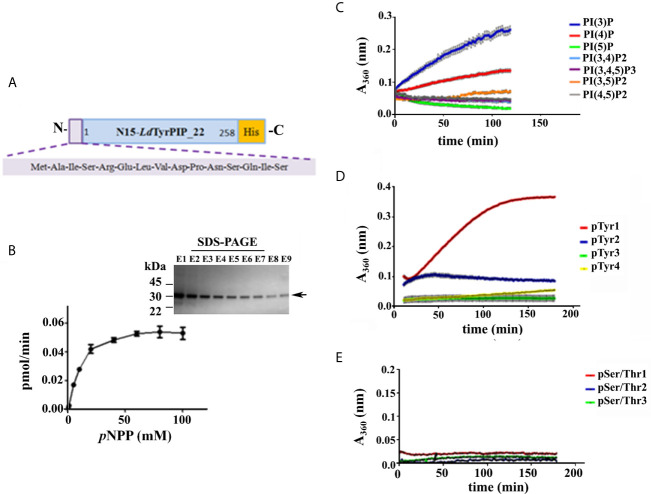
Study of the catalytic properties and substrate specificity of the *Ld*TyrPIP_22. **(A)** Schematic representation of the r*Ld*TyrPIP_22 protein expressed in bacteria for this study (281 aa, with calculated Molecular mass: 32,062). **(B)** Michaelis-Menten saturation curve for the enzymatic reaction of the rN15-*Ld*TyrPIP_22-His (2 *μ*M) using the *p*NPP (0.5–100 mM) as substrate. The reaction was performed at 25°C, pH 6. Inset: Coomassie stained SDS-PAGE of eluted fractions (E1-E9) from the purification of rN15-*Ld*TyrPIP_22-His from bacteria lysates (*Materials and Methods*) were analyzed on a 4%–12% gradient SDS-PAGE gel. Arrow on the right side indicates the expected position for migration of the rN15-*Ld*TyrPIP_22-His polypeptide; Time course of dephosphorylation of PI species (100 *μ*M) **(C)**, P-Tyr peptides (1 mM) **(D)**, and P-Ser/P-Thr peptides (60 *μ*M) **(E)**. Peptide aa sequences are shown in **Table S2**.

From the primary structure of *Ld*TyrPIP_22 was predicted that this protein must act as a PTP since it contains the characteristic P-loop motif sequence of the PTP catalytic center also described to be present in the sequence of the ALP enzyme family members (Beresford et al., 2010). To confirm the predicted PTP catalytic properties of *Ld*TyrPIP_22 we analyzed the enzyme kinetics and the substrate specificity of a *Ld*TyrPIP_22 recombinant form expressed in bacteria. For this we constructed two plasmids encoding the full length protein (residues 1–777) with a polyHis tag at the C-terminus. In the first plasmid the initiation of translation of the insert begun at the ATG codon of the *ldtyrpip__22_* sequence, whereas in the second plasmid translation was inititiated at the pTriEx1.1 plasmid’s ATG codon located 45 bp upstream the site where the *ldtyrpip__22_* sequence was inserted. The second construction encodes for a polypeptide with an additional 15-mer at the N-terminus (pTriEx1.1-N15-*ldtyrpip*__22_-His; **Figure 2A**). The recombinant protein produced from the first plasmid was mostly insoluble (**Figure S2A**). In contrast, the rN15-*Ld*TyrPIP_22-His polypeptide produced from the second plasmid (**Figure 2A**), was mostly soluble (**Figure S2B**). The rN15-*Ld*TyrPIP_22-His (281 aa, calculated MW 32062 Da), was subsequently purified by affinity chromatography on Ni^2+^ and/or Co^2+^ beads (**Figure 2B**, inset) and its enzymatic activity was assessed by monitoring the kinetics of dephosphorylation of the generic phosphatase substrate *p*NPP (**Table 1**, **Figure 2B**).

**Table 1:** Kinetic parameters of rN15-*Ld*TyrPIP_22-His (values are means ± S.E.M.).

Substrate	*K*m (mM)	*K*cat (s^-1^)	*K*cat*/K*m (M^-1^x s^-1^)
*p*NPP	11,793 ± 2,942	0,03124 ± 0,004	2.65 ± 0,280

**Figure 3:**
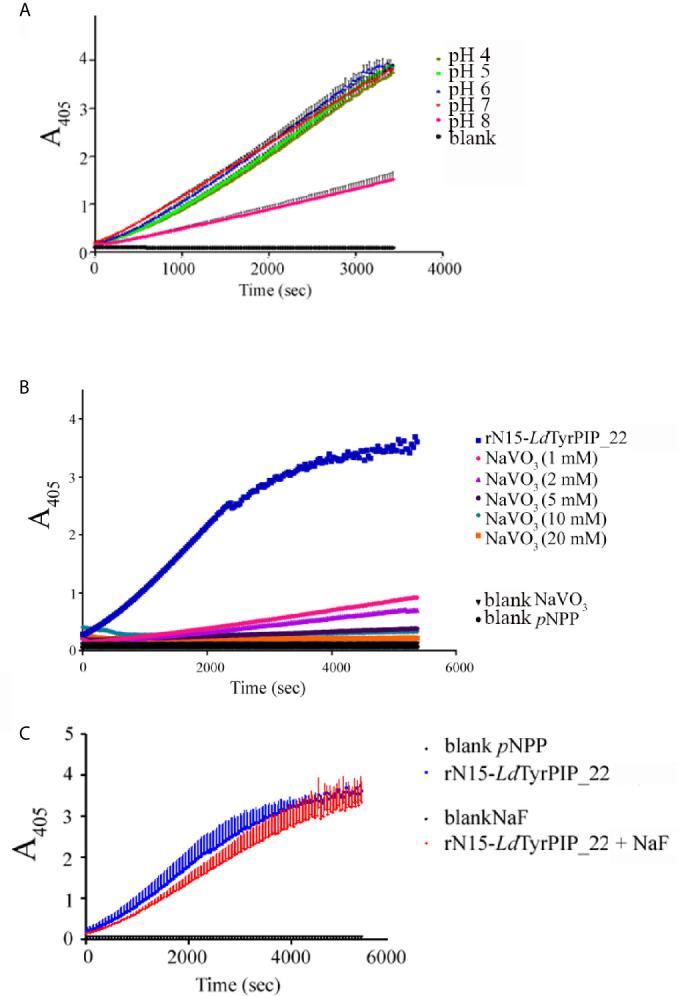
Effect of pH and inhibitors in the rN15-*Ld*TyrPIP_22-His phosphatase activity. The rN15-*Ld*TyrPIP_22-His phosphatase activity was assayed at 25°C (*Materials and Methods*) with *p*NPP (10 mM) as substrate, **(A)** in pH ranging from 4-8; **(B)** in the presence of the specific PTP inhibitor, Na_3_VO_4_ (1–20 mM) or **(C)** in the presence of the specific P-Ser/P-Thr phosphatase inhibitor, NaF (100 mM) at pH 6.

As expected, the rN15-*Ld*PIP22-His specific activity [74.8 ± 1.01 nmol *p*NP/min/mg (n=4)] estimated at 37°C and pH 6, was in the same range as the specific activity estimated for the *L. major* ortholog phosphatase (45.47 ± 1.52 nmol *p*NP/min/mg) reported in the original publication describing the ALP family in bacteria and lower eukaryotes (Beresford et al., 2010). Finally, the catalytic activity of rN15-*Ld*TyrPIP_22-His towards *p*NPP was shown to be optimal at pH 6 and 7 when measured at 25°C, while at 37°C the activity was found to be optimal at pH 6 (**Figures 3A** and **S3C**). Phosphatase activity was also detected at more acidic conditions (**Figures 3A** and **S3C**).

Apart from the *p*NPP substrate, we also compared the ability of rN15-*Ld*TyrPIP_22 to dephosphorylate a panel of PIs (**Figure 2C**) and peptides containing in their sequence P-Tyr or P-Ser/Thr (**Figures 2D, E**; **Table S3**). Our results indicated that rN15-*Ld*TyrPIP_22-His has a double substrate specificity. It dephosphorylated both monophosphorylated PIs [PI(3)P and PI(4)P] (**Figure 2C**) and P-Tyr containing peptides (**Figure 2D**), while it did not dephosphorylate any of the small number of P-Ser/P-Thr containing peptides tested (**Figure 2E** and **Table S2**). As control enzymes were used the bacterially expressed human phosphatase of regenerating liver-3 (PRL3) (Goldstein, 2001, Mc Parland et al., 2011) (**Figure S3A**) and the human protein-tyrosine phosphatase PTP1B (Wassef et al., 1985) (**Figure S3B**). The substrate selectivity of rN15-*Ld*TyrPIP_22 for P-Tyr was further confirmed by carrying out inhibition assays with the P-Tyr phosphatase inhibitor Na_3_VO_4_ and the P-Ser/P-Thr phosphatase inhibitor NaF using *p*NPP as substrate (10 mM) (**Figure 3**). Na_3_VO_4_ inhibited almost quantitatively the dephosphorylation of *p*NPP even at 1/10^th^ of the substrate concentration (**Figure 3B**), whereas NaF showed no inhibitory effect even at a concentration 10 fold higher than the substrate’s concentration (**Figure 3C**).

All the above described data confirmed that the recombinant bacterially expressed *Ld*TyrPIP_22 behaves as a dual specificity P-Tyr and PI phosphatase as expected from its sequence similarity to phosphatases from the ALP family (Beresford et al., 2010).

### *Ld*TyrPIP_22 Is Expressed in *L. donovani* Promastigotes as Soluble, Membrane Bound and Insoluble Cytoskeleton Associated Forms

**Figure 4:**
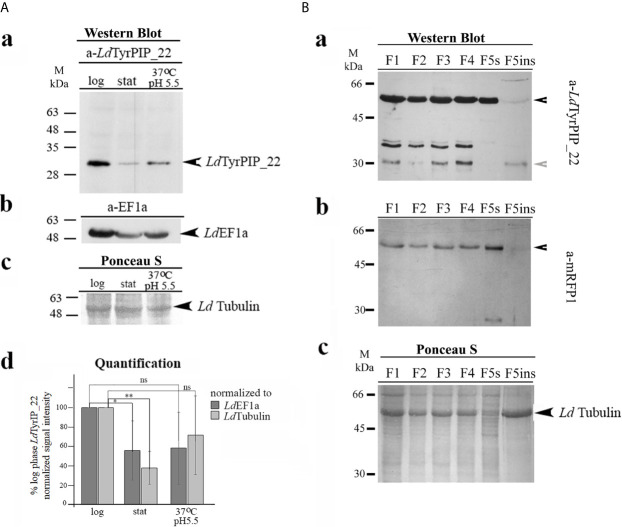
Biochemical detection of the endogenous *Ld*TyrPIP_22 forms in *wt L. donovani* (LG13) and in *L. donovani*-*ldtyrpip*__22_ *-mrfp1* transgenic promastigotes. Subcellular distribution of *Ld*TyrPIP_22 by detergent treatment. **(A)** Total lysates (40 μg protein/well) from logarithmic (log) and stationary (stat) phase cultures of promastigotes (~5X10^6^ cells), as well as from parasites after treatment of stationary phase promastigotes for 24 h at 37°C and pH 5.5, were analyzed by SDS-PAGE (12% w/v) and immunoblotted with the purified rabbit a-*Ld*TyrPIP_22 pAb (2 μg/ml) (a). The membrane was then rebloted after stripping with the a-EF1a mAb (1:10,000). *Ld*EF1a was used as loading indicator (b). The region of the membrane with tubulin stained with Ponceau-S is shown (c) as a 2^nd^ loading indicator. (d) Quantification of the intensity of the *Ld*TyrPIP_22 band signal for each condition was performed using the Fiji algorithm. The intensity of the *Ld*TyrPIP_22 band signal for each condition was normalized to the intensity of the corresponding *Ld*EF1a or *Ld*Tubulin band signals and expressed as % of the normalized *Ld*TyrPIP_22 signal at the logarithmic phase in each experiment. The mean values from three independent experiments with standard deviations were plotted. ** (P ≤ 0.01), * (P ≤ 0.05) and ns (P > 0.05). **(B)** Protein fractions (F1-F5ins) from stationary phase *L. donovani*-*ldtyrpip*__22_ *-mrfp1* transgenic promastigotes, produced by permeabilization of total cell pellet with digitonin and Triton X-100 (*Materials and Methods*) were analyzed by SDS-PAGE (12% w/v) and immunoblotted with the mouse a-*Ld*TyrPIP_22 serum (1:1,200) (a) and subsequently, after stripping, with the purified a-mRFP1 (0.5 μg/ml) pAb (b). Ponceau-S of respective membrane regions (c), is shown as loading indicator. Black arrowhead indicate the protein bands corresponding rabbit to r*Ld*TyrPIP_22-mRFP1 and grey arrowhead to band assigned to the endogenous *Ld*TyrPIP_22. Molecular weights are indicated in kDa.

To confirm the expression of the *LDBPK_220120.1* encoded *Ld*TyrPIP_22 protein in *L. donovani* cells and assess its abundance at different developmental stages of the parasite we used axenic *L. donovani* promastigote cultures growing at 25°C and pH 7 at the logarithmic (enriched in dividing cells) and stationary (enriched in non-dividing cells/metacyclis) phases of growth. We also used stationary phase promastigotes cultured for another 24 h at 37°C and pH 5.5, conditions mimicking the temperature and pH of the parasitophorous phagolysosome’s environment in mammalian macrophages (Mc Parland et al., 2006). For this, total lysates from these parasites were analyzed by Western Blot using a-*Ld*TyrPIP_22 specific polyclonal antibodies (pAbs) generated for this project (*Materials and Methods* and **Figure S4A**). The rabbit pAb detected in all three cases [i.e., logarithmic and stationary phase promastigotes growing at 25°C and pH 7 and promastigotes subjected to heat (37°C) and low pH (5.5) treatment] a protein species with apparent MW ~ 30 kDa (**Figure 4Aa**). Given that the calculated MW of *Ld*TyrPIP_22 is 29.1 kDa we consider that this ~30 kDa protein species corresponds to the nascent *Ld*TyrPIP_22 form. Occasionally we also detected a pair of bands migrating with lower mobility below the 35 kDa protein size marker (**Figure S4B**). Quantification of the signal intensity of the ~ 30 kDa band detected by the specific a-*Ld*TyrPIP_22 rabbit pAb showed significantly higher levels in the logarithmically growing cells than in the stationary phase ones (**Figure 4Ad**).

To examine whether the *Ld*TyrPIP_22 protein could preferentially associate with certain subcellular compartments, we prepared subcellular fractions from transgenic *L. donovani* promastigotes expressing a chimeric *Ld*TyrPIP_22-mRFP1 which would serve as an internal positive control for the specificity of the a-*Ld*TyrPIP_22 pAbs. The biochemical subcellular protein fractionation performed was based on a protocol using a stepwise permeabilization of promastigote membranes by incubating with gradually increasing concentrations of the natural detergent digitonin (Foucher et al., 2006) followed by an additional membrane solubilization step in which the final pellet [Fraction 5 (F5)] was treated with 1% (v/v) Triton X-100 [(Doukas et al., 2019), *Materials and Methods*]. This procedure allowed preparation of subcellular fractions enriched in soluble cytosolic proteins (Fractions F1 and F2), proteins associated with intracellular organelles (Fraction F3 and F4), endoplasmic reticulum (ER) (Fraction F4), pelicular membrane (Fraction F5s), and an insoluble fraction (F5in, final insoluble pellet) enriched in nuclear/cytoskeleton proteins. We further analyzed these fractions by WB using the mouse a-*Ld*TyrPIP_22 pAb that we generated (**Figure S4A**).

A band detected with apparent MW ~ 55 kDa (**Figure 4Ba**) probably corresponds to the recombinant *Ld*TyrPIP_22-mRFP1 polypeptide (calculated MW 54.5 kDa) since it was also detected by the a-mRFP1 specific pAb (**Figure 4Bb**). Three more protein species were detected at the 30-35 kDa range with the a-*Ld*TyrPIP_22 pAb (**Figure 4Bc**), not recognized by the a-mRFP1 pAb (**Figure 4Bb**). The lower size band of ~30 KDa most probably corresponds to the endogenous *Ld*TyrPIP_22 form identified in the wt *L. donovani* total lysates by the rabbit a-*Ld*TyrPIP_22 pAb (**Figure 4Aa**). The doublet just below 35 kDa could be assigned to proteolytic fragments of the *Ld*TyrPIP_22-mRFP1 not recognized by the a-mRFP1 pAb under the specific conditions of the experiment. Interestingly, the recombinant full length *Ld*TyrPIP_22-mRFP1 was detected in all subcellular fractions analyzed [F1-F5ins]. The three protein species of lower size were detected in all fractions except the F5s (**Figure 4Bc**) a fraction shown before (Doukas et al., 2019) to be enriched in pelicular membrane proteins. The presence of *Ld*TyrPIP_22-mRFP1 in the F5s fraction could represent a translocation artifact due to overexpression, as previously observed for other chimeric proteins tagged with fluorescent proteins (Moore and Murphy, 2009). Finally, a small fraction of the ~ 30 kDa species, assigned to the endogenous *Ld*TyrPIP_22 and of the full length *Ld*TyrPIP_22-mRFP1 were faintly detected in the F5ins fraction which is enriched in nuclear/cytoskeletal proteins (Foucher et al., 2006). Monomeric free mRFP1 (MW ~27 kDa), probably produced by proteolysis of the full length chimera *Ld*TyrPIP_22-mRFP1, was only detected in fraction F5s (**Figure 4Bc**). In previously published work we have shown that free mRFP1^9^ produced in transgenic *L. tarentolae*-mRFP1 cells was mostly recovered in fractions F1 and F2 whereas ER-associated or pelicular membrane proteins were recovered in fractions F4 and F5s (Papadaki et al., 2015; Doukas et al., 2019). It is worth emphasizing that both a-*Ld*TyrPIP_22 Abs (mouse and rabbit) detected the 30 kDa protein species (**Figures 4Aa, Ba**), a result that strengthens the assignment of this band to the endogenous *Ld*TyrPIP_22 protein.

Since there is evidence for secretion of the bacteria ALP homologs of *Ld*TyrPIP_22 (i.e., MptpB and LipA) (Koul et al., 2000; Kastner et al., 2011), we also investigated whether the *Ld*TyrPIP_22 could be secreted by *wt L. donovani*. For this, we analyzed by WB the proteins in the extracellular medium of axenic *L. donovani* promastigotes growing at 25°C (temperature of the invertebrate host) at the stationary phase of growth or at 37°C (temperature of the mammalian host) following the two approaches described in the *Materials and Methods*. Despite repeated attempts we were not able to detect the *Ld*TyrPIP_22 amongst the proteins collected from the extracellular medium of the *L. donovani* LG13 strain incubated without serum for 9 h at 25°C or for 6 h at 37°C (data not shown). These assays did not include however the challenging analysis of proteins that would be released by the amastigotes inside the infected macrophages.

Thus, the *Ld*TyrPIP_22 protein, encoded by the *LDBPK_220120.1* gene, is expressed in *L. donovani* promastigotes at the logarithmic and stationary phases of growth, as well as in promastigotes subjected to heat (37°C) and pH (5.5) stress for 24 h. Interestingly, the *Ld*TyrPIP_22 was detected in several different subcellular fractions enriched in cytoplasmic, membrane or cytoskeleton associated proteins.

### *Ld*TyrPIP_22 Is Localized in Multiple Sites in *L. donovani* Promastigotes Growing at 25°C and pH 7

To study further the *Ld*TyrPIP_22 subcellular distribution in *Leismania* cells we performed immunofluorescence labeling and confocal microscopy analysis using our homemade mouse and rabbit a-*Ld*TyrPIP_22 pAbs (**Figure S4A, B**) in combination with Abs recognizing other *Leishmania* proteins of known subcellular localization, including *Ld*Actin (Sahasrabuddhe et al., 2004), *Ld*Tubulin, and GAPDH as a glycosome marker (Herman et al., 2008).

We first analyzed the *Ld*TyrPIP_22 localization in axenic dividing and non-dividing promastigotes grown at 25°C using logarithmic or stationary phase promastigote cultures. Having at our disposal a-*Ld*TyrPIP_22 Abs from two species, allowed us: 1) to ensure that the epitopes highlighted by each Ab belong to the endogenous *Ld*TyrPIP_22 (**Figure S4B**) and 2) to perform co localization studies with other *Leishmania* proteins using specific primary Abs either from rabbit or mouse origin. For imaging of the parasites besides the TCS SP Leica confocal microscope we used the SP5 and SP8 Leica more advanced confocal models which allowed us to benefit from maximum optical resolution of the lenses and higher digital resolution in the image acquisition than the older TCS SP confocal microscope.

**Figure 5:**
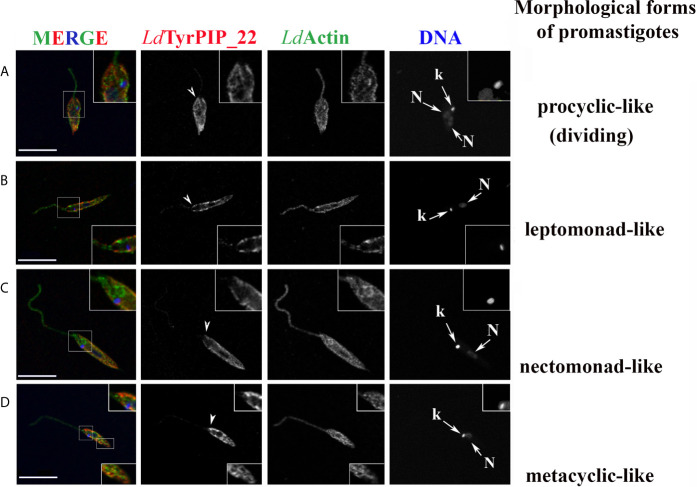
Localization of *Ld*TyrPIP_22 in different cell cycle dependent morphological forms of cultured *L. donovani* promastigotes growing at 25°C, pH 7; co staining for *Ld*Actin. *L. donovani* promastigotes from logarithmic or stationary phase cultures were fixed (4% w/v PFA) and permeabilized with 0.1% (v/v) TritonX-100. They were then co stained by IF with the a-*Ld*TyrPIP_22 mouse pAb (1:250) and rabbit a-LeishActin pAb (1:1,000) followed by secondary Abs conjugated to Alexa Fluor^®^546 and Alexa Fluor^®^ 488, respectively. Nuclear and kinetoplast DNA was stained with Hoechst 33342 (1:5,000). Representative images of different cell cycle dependent morphological forms of promastigotes were acquired by z-scanning performed at 0.3 *μ*m step size using the TCS SP8 Leica confocal microscope. A single z section with representative staining from each case is shown. Single FL images are shown in black and white (BW) for better contrast while images of the merged FL signals are shown in color. The molecule highlighted in the BW images with single color FL is indicated at the top in the same color as the respective FL signal. Arrowheads point to the flagellar pocket region/flagellum base. Small arrows point to the kinetoplast (k) and nucleus (N). Magnifications (1.5-2X) of framed areas are shown as insets. Scale bar size: 10 *μ*m.

The localization pattern of *Ld*TyrPIP_22 varied in parasites at different cell cycle dependent morphological stages (**Figure 5** and **Figure S5**). Depending on the cell cycle stage, axenic *Leismania* promastigotes obtain diverse morphologies most of which, with exception the procyclic and the metacyclic forms, coexist both in the exponential and stationary phases of growth (Wheeler et al., 2011). These morphologies, following nomenclature used for description of the flagellated promastigote forms observed in the midgut of sandflies (Sunter and Gull, 2017), could be assigned as: 1) dividing procyclic-like with short cell body (6.5–11.5 μm) and flagellum shorter than the cell body; 2) leptomonad-like with cell body between 6.5–11.5 μm and longer flagellum; 3) nectomonad-like with cell body longer than 12 μm and long flagellum and 4) the metacyclic-like with short (8 μm) and slender cell body and long flagellum (>2X longer than the cell body).

Interestingly, in dividing and non-dividing morphological forms of promastigotes in culture, *Ld*TyrPIP_22 was localized at the periphery of the cell body (**Figure 5**, **Figures S5Ac, d**, **S6** and **S9**) in areas rich in *Ld*Actin (**Figures 5A–D** insets) and microtubules (**Figures S6**) or stained with the FM4-64 dye (**Figure S7**) (Vince et al., 2008). Occasionally, *Ld*TyrPIP_22 epitopes were also detected in internal tubular/filament-like structures (**Figure 5D**, bottom inset, **Figures S5Ab**, **S7b**) which could be part of the tubular MTV endosome (Halliday et al., 2019; Wang et al., 2020) and at the posterior region of the cell body (**Figure 5A**, **Figure S7b**), area where internalized/endocytosed cargo accumulates in late endosomes (Ghedin et al., 2001; Fernandes et al., 2020). Lower intensity punctate staining was also observed along the entire flagellum in most parasites (**Figures 5B** and **S5Aa, c, d** and **S5Bb**). In this analysis, *Ld*Actin was detected both at the promastigote’s cell body and along the flagellum with the labelling of the a-*Ld*Actin Ab more evident at the pepriphery of the cell body including the area where the flagellum buds. The pellicular membrane-associated actin has been described to be closely localized with the subpellicular microtubules (sMTs) (Sahasrabuddhe et al., 2004). Consistently, co staining with the *Ld*TyrPIP_22 rabbit pAb and a mouse Ab for *Leishmania* tubulin, highlighting the subpelicular microtubules, confirmed the localization of *Ld*TyrPIP_22 at the periphery of the promastigote’s cell body (**Figure S6**) either subpelicularly or at the pelicular membrane.

Given the limitation of the optical resolution of confocal microscopes we cannot ascertain protein co-localization or distinguish between the subpelicular actin or microtubules and the pelicular membrane. However, quantification of the % of red pixels in the cell body (corresponding to *Ld*TyrPIP_22 epitopes), overlapping with the green pixels (corresponding to *Ld*Actin epitopes), resulted in an estimation of 59 ± 14% (n=49) of *Ld*TyrPIP_22 signal superimposed with the *Ld*Actin signal in all the morphological forms of promastigotes from a promastigotes’ culture. Higher levels of pixel co-localization were observed in procyclic-like/dividing and metacyclic-like parasites (60 ± 12, 63 ± 10 and 65 ± 11% respectively) while for the leptomonad- and nectomonad-like forms the overlapping pixels ranged from 50 ± 11-52 ± 14%. Therefore, from this pixel intensity analysis, a molecular proximity of *Ld*TyrPIP_22 with *Ld*Actin is inferred with the possibility of a direct or indirect interaction.

Additional sites where *Ld*TyrPIP_22 was detected in axenic promastigote, regardless the a-*Ld*TyrPIP_22 pAb used were: 1) next to or surrounding the kinetoplast (the mass of concatenated mitochondrial DNA) (**Figure S5**, arrowheads), 2) the base or the sides of the flagellar pocket (invagination of the cell membrane forming a vase-like structure at the base of the flagellum) and 3) the proximal end of the flagellum (**Figures 5** and **S5**, short arrowheads). Often the entire flagellar pocket was decorated by the a-*Ld*TyrPIP_22 Ab staining (**Figures S5Ac** and **S5Ba,b** insets). In newly divided (**Figure S5Ac**) and dividing promastigote, identified by the two nuclei (**Figure S5Ad**), we also observed staining of vesicular-like structures. Co staining with the a-GAPDH antibody, GAPDH was used as a glycosome marker, showed that the *Ld*TyrPIP_22 vesicular staining was excluded from the structures highlighted by this Ab (**Figure S8**). The punctate staining observed around large compartments (**S5A** **a** inset, **c**) could mark acidocalcisomes or megasomes (Rodrigues et al., 2014).

Overall, *Ld*TyrPIP_22 epitopes were detected in multiple sites in the *L. donovani* axenic promastigote with the most prominent ones being 1) the periphery of the cell body, 2) adjacent to the kinetoplast, 3) intracellular small vesicles, 4) the proximal end of the flagelum, and 5) the flagellar pocket area.

### A Shift to 37°C and pH 5.5 Triggered a Pronounced Recruitment of *Ld*TyrPIP_22 at the Flagellar Pocket Region and a Perinuclear Redistribution

In the *in vivo* situation, the differentiation process converting the *Leishmania* promastigote to amastigote takes place within the mammalian host macrophages when the infective metacyclic promastigote is phagocytozed and enclosed in the parasitophorous phagolysosome (Sunter and Gull, 2017). The metacyclic promastigote (MP) to the amastigote transition involves dramatic changes in cell shape and results in a minimized cell surface to volume ratio, hence reducing the area over which the cell is exposed to the harsh environment of the parasitophorous vacuole reformatting of the flagellum and also occurs. These changes require substantial membrane and cytoskeleton remodeling in which, from what is known for higher eukaryotes (Schink et al., 2016; Senju et al., 2017), PI metabolism and signaling play central roles. Therefore, we decided to monitor by microscopy the localization of the *Ld*TyrPIP_22 over time under conditions resembling those experienced by the promastigote when enclosed in the parasitophorous phagolysome.

**Figure 6:**
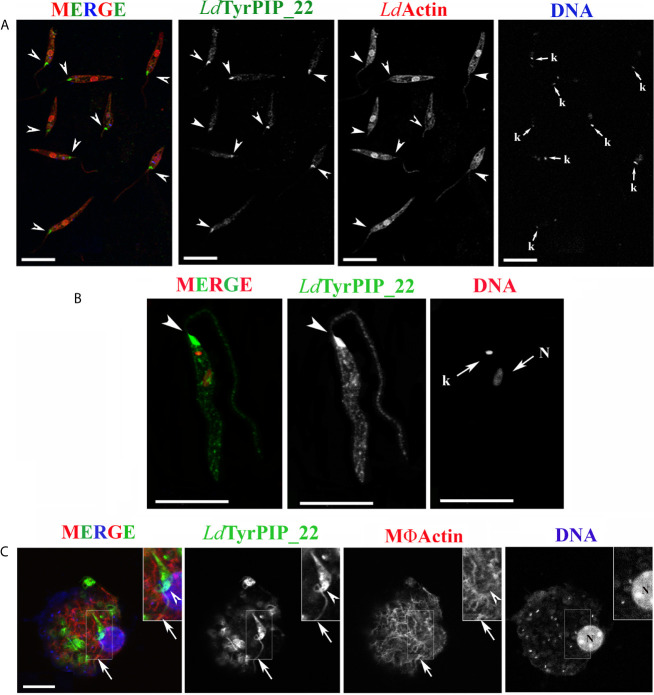
Localization of *Ld*TyrPIP_22 epitopes in axenic *L. donovani* promastigotes after 24 h exposure at 37°C and pH 5.5 or during a 4 h *in vitro* infection of J744 macrophages. **(A, B)** Confocal microscopy images of *L. donovani* stationary phase promastigotes incubated for 24 h at 37°C and pH 5.5. Images were acquired with a Leica SP8 confocal microscope by z-scanning with 0.3 μm step size. Images in **(A)** were acquired at 1X digital magnification while in B at 4X. **(A)** Double IF staining of fixed cells permeabilized with 0.1% (v/v) Τriton X-100 was performed with the a-*Ld*TyrPIP_22 mouse pAb (1:100) and a-*Ld*Actin rabbit pAb (1:1,000) followed by secondary Abs conjugated to Alexa Fluor ^®^ 488 or Alexa Fluor ^®^ 546. Nuclear and kinetoplast DNA was stained with Hoechst 33342 (1:5,000). Images (max projections) of several promastigotes are presented. In the DNA stain the kinetoplast (small arrow, k) is more intensely stained than the nucleus. **(B)** An image of a promastigote (single z section of a z-series) acquired with a 4X magnification factor. IF staining for *Ld*TyrPIP_22 and DNA staining were performed as in **(A)**. The nuclear (N) and kinetoplast (k) DNA staining is presented here in red pseudo color for better contrast in merged image. **(C)** A J774 murine macrophage 4 h post-infection *in vitro* with *L. donovani* promastigotes imaged by confocal microscopy. Stationary phase promastigotes were added to adhered macrophages at a 20:1 ratio. IF staining of fixed cells permeabilized with 0.1% (v/v) Τriton X-100 was performed with the a-*Ld*TyrPIP_22 mouse pAb (1:100) followed by secondary Ab conjugated to Alexa Fluor ^®^ 488. Macrophage polymerized actin was stained with phalloidine conjugated to Alexa Fluor ^®^ 546. Images were acquired with a Leica TCS SP5 confocal microscope by z-scanning performed at 1 μm step size. A single optical section from the top of the cell is presented. A promastigote, still extracellular in most part but in the process of being phagocytozed is shown in the insets (1.5X magnification). Single FL and phase contrast images are presented in BW while images of the three merged FL signals are shown in color. The molecule highlighted in the BW images with single color FL is indicated at the top in the same color as the respective FL signal. In **(A)**, **(B),** and **(C)** arrowheads point to the flagellar pocket/base of flagellum. Small arrows in **(A)** and **(B)** point to the kinetoplast (k) and nucleus (N) while large arrows in **(C)** point to polymerized actin surrounding part of the phagosytozed parasites’ flagellum. Scale bar size in **(A–C)**: 10 μm.

**Figure 7:**
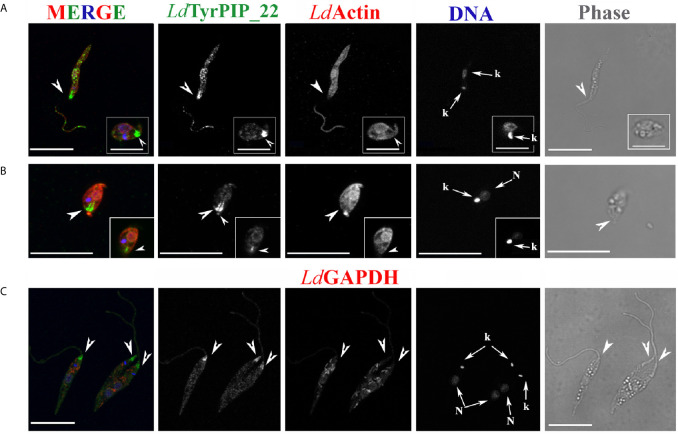
Localization of *Ld*TyrPIP_22 epitopes in tationary phase *L. donovani* parasites after 24 h exposure at 37°C and pH 5.5. Co staining with *Ld*Actin and *Ld*GABDH. Confocal microscopy images of *L. donovani* promastigote- and amastigote–like forms. Double IF staining of fixed cells permeabilized with 0.1% (v/v) TritonX-100 was performed with the anti-*Ld*TyrPIP_22 mouse pAb (1:100) and the anti-*Ld*Actin rabbit pAb (1:1,000) in **(A, B)** or the anti-GAPDH rabbit pAb (1:100) in **(C)** followed by secondary Abs conjugated to Alexa Fluor ^®^ 488 or Alexa Fluor ^®^ 546. Nuclear and kinetoplast DNA was stained with Hoechst 33342 (1:5,000). In **(A, C),** single z-sections of axenic promastigote-like forms. Inset in **(A)**: an amastigote-like parasite from the same field at a 2X magnification. In **(B),** the maximum intensity projection of an amastigote-like parasite (i.e., smaller than promastigotes, ovoid, with retracted/short flagellum). Inset in **(B)**: a single z-section at the same magnification as the maximum intensity projection. Single FL and phase contrast images are presented in BW while images of the three FL signals merged are shown in color. The molecule highlighted in the BW images with single color FL is indicated at the top in the same color of the respective FL signal. Images were acquired with a Leica TCS SP8 confocal microscope by z-scanning performed at 0.3 μm step size. Large arrowheads point to the flagellar pocket/base of flagellum. Small arrowhead in inset **(B)**, shows the small flagellum. Small arrows in **(A–C)** point to the kinetoplast (k) and nucleus (N). Scale bar size in A-C: 10 μm. Inset **(A)** scale bar size: 5 μm.

To this end, a stationary phase promastigote culture, 8 days post subculturing, was subjected to a temperature shift at 37°C while the pH was adjusted to 5.5. The conversion of the promastigotes’ population to amastigotes in most protocols begins 4 h after the temperature and pH shift. In the first 24 h of incubation of the axenic parasites’ culture at 37°C and pH 5.5, a mixed population of flagellated promastigotes and amastigote-like parasites with short flagella coexist. (Doyle et al., 1991; Debrabant et al., 2004; Zilberstein, 2020). Imaging of promastigotes from this population using immunofluorescence and confocal microscopy showed a systematic and drastic recruitment of the *Ld*TyrPIP_22 towards the base of the flagellum and the flagellar pocket (**Figures 6** and **7**). The other two prominent localizations observed were: 1) a perinuclear punctate staining (**Figures 6A**, **7A–C**) and 2) a vesicular staining throughout the cell body (**Figure 6B**). Of note, in this axenic promastigote culture we also observed *Ld*Actin in the nucleus (**Figures 6A**, **7A, B**) as it has already been reported (Sahasrabuddhe et al., 2004).

To refine the conditions that induce accumulation of the *Ld*TyrPIP_22 at the promastigote’s flagellar pocket, we tested separately the effect of temperature and pH. We found that as early as 30 min after transferring the promastigote to 37°C we could detect the distinct accumulation of *Ld*TyrPIP_22 at the promastigote’s flagellar pocket (**Figure S7**, arrowhed). A similar *Ld*TyrPIP_22 distribution was observed in extracellular promastigotes in an *in vitro* infection assay of J744 murine macrophages exposed only to 37°C but not to low pH shift 4 h post-infection (**Figure 6C**, insets).

In this case the promastigotes were promastigotes grown at 25°C and pH 5.5 for 24 h did not show this characteristic recruitment of *Ld*TyrPIP_22 at the flagellar pocket (data not shown). Moreover, in amastigote-like forms, observed in the axenic culture grown at 37°C and pH 5.5 for 24 h up to 144 h, *Ld*TyrPIP_22 epitopes were detected at the flagellar pocket near the kinetoplast (**Figures 7A** inset, **7B** and **S9B**), in vesicles (**Figure S9A**) or at the tip of the small flagellum barely emerging from the flagellar pocket (**Figures 7B** and **S9B**).

Concluding, at 37°C and pH 5.5, conditions resembling the ones encountered by *Leishmania* parasites in the mammalian hosts where the developmental transition of the MPs to amastigotes is initiated, *Ld*TyrPIP_22 is strongly recruited to the flagellar pocket and redistributes perinuclearly.

## Discussion

This study provides the first experimental evidence documenting the expression of the *L. donovani LDBPK_220120.1* gene product in the *L. donovani* parasites, a *Leishmania* spp. highly pathogenic to humans. The ortholog gene *LmjF_22_0250* from *L. major*, shown to be upregulated in metacyclic *L. major* promastigote (Dillon et al., 2015; Inbar et al., 2017), was earlier reported to encode a protein which when expressed as recombinant in bacteria exhibited PTP and PI phosphatase activities (Beresford et al., 2010).

We cloned the *LDBPK_220120.1* gene from a Sudanese *L. donovani* (LG13) strain and showed that its protein product, when expressed heterologously in bacteria, dephosphorylated P-Tyr peptides and monophosphorylated PIs [PI(3)P and PI(4)P], similarly to its *L. major* ortholog LM1 (Beresford et al., 2010), properties that classify both as atypical Dual Specificity Lipid-like phosphatases (Brenchley et al., 2007). The *L. donovani* aDSP, that we named *Ld*TyrPIP_22, together with its *L. major* ortholog, represent to our knowledge the first PI phosphatases characterized in *Leishmania* spp. to date. With specific antibodies that we generated we analyzed the subcellular localization of *Ld*TyrPIP_22 using detergent based subcellular fractionation and immunofluorescence microscopy. An array of intracellular localizations, comprising membrane and cytoskeleton associated elements, were observed by both approaches, suggesting that *Ld*TyrPIP_22 has multiple cellular partners/substrates participating thereby in multiple cellular functions which are consistent with its identity as a dual specificity P-Tyr/PI phosphatase.

Interestingly, the recombinant *Ld*TyrPIP_22 used in this study has a narrower substrate preference than its bacterial homologs MptpB and LipA from the ALP family (Beresford et al., 2010). MptpB from *Mycobacterium tuberculosis* has triple specificity, dephosphorylating P-Ser, P-Thr, and P-Tyr peptides as well as mono- and di-phosphorylated PIs (i.e., PI(3)P, P(4)P, PI(5)P, and PI(4,5)P*_2_*), (Beresford et al., 2010). LipA, from *Listeria monocytogenes* although it has similar substrate preference to the PIs with MptpB, it dephosphorylates only P-Tyr peptides (Beresford et al., 2010). The P-loop sequence in the catalytic site of the three proteins is almost identical with only one aa difference at position 2 (i.e., Phe^161^ in MptpB instead of Thr^148^ in r*Ld*TyrPIP_22 and Thr^188^ in LipA). Point mutation studies in the MptpB (Beresford et al., 2007) showed that the nature of this aa plays an important role in the substrate specificity for PIs as well as in the levels of enzymatic activity. However, other determinants in the 3D structure of the three proteins must play critical role in the substrate preference. The bacterially expressed ortholog of *Ld*TyrPIP_22 from *L. major* (i.e., LM1), additionally to the PI(3)P and PI(4)P, was shown to also dephosphorylate PI(5)P (Beresford et al., 2010). The predicted primary structures of the two proteins differ in 7 aa (**Figure 1**). Whether these differences play a critical role on the two enzymes’ substrate preferences remains to be shown.

Phosphorylation and dephosphorylation are well-documented post translational modifications regulating the developmental transitions of *Leishmania* spp. along their biological cycle (Chow et al., 2011; Tsigankov et al., 2013; Tsigankov et al., 2014; Dillon et al., 2015). Especially, phosphorylation on tyrosine residues, although comprising a small fraction of all protein phosphorylation events, plays a pivotal role in signaling, cell-cycle control, and differentiation (Cool and Blum, 1993; Nascimento et al., 2006). Indeed, inhibition of tyrosine phosphorylation in *Leishmania* promotes partial differentiation from promastigote to amastigote forms (Nascimento et al., 2003). Therefore tyrosine kinases and P-Tyr phosphatases can be viewed as master regulators of the *Leishmania* developmental cycle. The *Ld*TyrPIP_22 phosphatase represents a putative player in these regulations with its P-Tyr phosphatase activity. The finding that the *LDBPK_220120.1* ortholog gene transcription in *L. major* is significantly upregulated in both cultured and sandfly metacyclic promastigotes (Dillon et al., 2015; Inbar et al., 2017) suggests a need for this enzyme’s activity over the developmental transition of *Leishmania* from the forms adapted in the blood feeding insects’ midgut/foregut to the ones living in the mammal dermis and skin-distant organs. Moreover, the substantial upregulation (5.92 fold vs 2.63 fold) of this *L. major* transcript in the sandfly living metacyclics (Inbar et al., 2017) versus the cultured metacyclis (Dillon et al., 2015), points to its importance in the survival of the parasite under the stress and nutrient scarcity conditions in the anterior midgut of the fly where metacyclogenesis occurs. Moreover, the MP to the amastigote transition involves dramatic changes in cell size and shape and a reformatting of the flagellum. These changes require substantial membrane and cytoskeleton remodeling and dynamics in which, from what is known for higher eukaryotes (Schink et al., 2016; Senju et al., 2017), PI signaling and metabolism play central roles. *Ld*TyrPIP_22 with its PI dephosphorylating activity may contribute to events resulting in these developmental transitions of *Leishmania* promastigotes.

In this study, the microscopy imaging of promastigotes growing at 25°C revealed *Ld*TyrPIP_22 epitopes at the flagellar pocket region (**Figures S5A** small arrows, **B** and **Ba,b** insets), adjacent to the kinetoplast (**Figure S5A** arrowheads) where the Golgi apparatus is located (Halliday et al., 2019) and occasionally in vesicular (**Figure S5A**), and tubular structures (**Figures S5**b and **S7**b) resembling MTV endosomes (Wang et al., 2020). All these localizations, considering that 1) the flagellar pocket region in protozoans of the *Leishmania* genus is highly specialized for uptake of macromolecular nutrients and secretion *via* the conventional exocytic route (Landfear and Ignatushchenko, 2001) and 2) the *Ld*TyrPIP_22 dephosphorylates selectively PI(3)P and PI(4)P, are compatible with a role for this phosphatase in endocytosis/exocytosis event(s) and/or membrane trafficking in *Leishmania* promastigotes. Diverse functions of PI(3)P and PI(4)P PIs in the regulation of membrane traffic and cell signaling pathways are based, at least in part, on their spatiotemporally controlled formation and turnover at defined subcellular sites (Marat and Haucke, 2016). In metazoan cells, PI 3-phosphates such as PI(3)P and PI(3,5)P_2_ are found predominantly in membranes of early and late endosomes or lysosomes with key importance for their function (Balla, 2013). Endosomal recycling to the cell surface requires PI(3)P hydrolysis and the concomitant generation of PI(4)P is required for exocytosis to occur (Wallroth and Haucke, 2018). PI(4)P is mainly concentrated in the exocytic pathway, in particular in the Golgi complex, the trans-Golgi network (TGN), and the plasma membrane (Balla, 2013; Hanes et al., 2017; Wallroth and Haucke, 2018). However, the *Leishmania* cell is largely an unmapped territory with respect to its PIs’ localization, metabolism and signaling pathways (Zhang and Beverley, 2010; Velasquez et al., 2015). In *Trypanosoma brucei*, one of the closest relatives of *Leishmania* spp. in the Trypanosomatidae family, it is known that PIs and their related kinases and phosphatases function as a regulatory system in addition to their role in the synthesis of membrane or glycoconjugate structures (Cestari, 2020). This is evidenced by the numerous cellular processes affected when genes encoding PI-related proteins or proteins generating PI metabolites are mutated or knocked out (reviewed in (Cestari, 2020)). Interestingly, in *Trypanosoma brucei*, PIs have diversified in function with respect to their role in metazoans to control specialized processes (Cestari and Stuart, 2018; Cestari et al., 2019). If we extrapolate from the localizations of PI(3)P and PI(4)P in metazoans and what is known for *Trypanosoma brucei, Ld*TyrPIP_22 may be involved in endocytosis/exocytosis event(s) and/or membrane trafficking in *Leishmania* promastigotes.

The distinct, although not prominent, detection of *Ld*TyrPIP_22 epitopes along the flagellum could support the hypothesis that this enzyme may also be involved in the regulation of intraflagellar transport or the flagellum’s remodeling during MP to amastigote differentiation. This could be another explanation for the *Ld*TyrPIP_22 epitopes detected to sites adjacent to the kinetoplast, known to be connected *via* a tripartite attachment complex (TAC) to the flagellum basal body (Gluenz et al., 2010). Recent studies on mammalian epithelial cells have shown that PI(4)P homeostasis, coordinated by a pair of PI kinase and phosphatase at the centrosome/ciliary base, is vital for ciliogenesis (Xu et al., 2016). It is possible that a similar mechanism also exists in *Leishmania* cells.

Finally, the observed localization of *Ld*TyrPIP_22 in regions enriched in *Ld*Actin, mainly at the periphery of the cell body of promastigotes growing at 25°C (**Figure 5**), suggests a possible role of this phosphatase in the regulation of actin cytoskeleton properties, as it is the case for a number of other phosphatases in lower (Delorme et al., 2003; Schuler and Matuschewski, 2006; Fernandez-Pol et al., 2013) and higher eukaryotes (Larsen et al., 2003; Nakashima and Lazo, 2010). From the quantitative analysis on the microscopy images of promastigotes co stained with the a-*Ld*TyrPIP_22 and the a-*Ld*Actin specific Abs, a molecular proximity of these two proteins was inferred with the possibility of a direct or indirect interaction. This could drive a working hypothesis on a dynamic interplay between the *Ld*TyrPIP_22 phosphatase and actin *via* its PI or its P-Tyr dephosphorylating activities. Interestingly, actin controls the motile behavior of the *Leishmania* promastigote and is involved in intracellular vesicular trafficking and flagellar-pocket organization in *Trypanosomatids* (Sahasrabuddhe et al., 2004; Katta et al., 2010). Both functions are compatible with the *Ld*TyrPIP_22 localizations in the *L. donovani* promastigotes.

One of the most novel findings of this study was the pronounced recruitment of *Ld*TyrPIP_22 in the flagellar pocket region when stationary phase promastigotes were subjected to temperature 37°C and pH 5.5 for 24 h (**Figures 6**, **7**). This accumulation did not seem to be due to higher expression levels of *Ld*TyrPIP_22 since quantification by Western Blot of the *Ld*TyrPIP_22 protein levels in axenic *L. donovani* promastigotes treated as above did not show a significant difference with the levels observed in stationary phase promastigotes growing at 25°C and pH 7 (**Figures 4Ba** and **d**). Environmental signals like elevated temperature and acidity have already been shown to be crucial in triggering the developmental transition from the motile MP to the immotile amastigote inside the mammalian host macrophage (Zilberstein and Shapira, 1994; Sereno and Lemesre, 1997; Barak et al., 2005). This differentiation marked by a dramatic change in the parasite’s cell shape from the elongated slender cell body to a small ovoid shape one and from a long motile flagellum with a 9 + 2 axoneme arrangement to a short non motile flagellum with a 9 + 0 axoneme arrangement (Gluenz et al., 2010). Additionally, a large restructuring of the flagellar pocket and neck region takes place. This associates with changes in the localization of the flagellum attachment zone proteins. As a consequence, the flagellar pocket neck closes to fit tightly around the remaining short flagellum end (Sunter and Gull, 2017) the tip of which is considered to play a sensory role in the interaction with the host (Kelly et al., 2020). Moreover, the growth rate and the metabolism of the amastigotes slow down considerably (McConville et al., 2007) by the expression of stage specific survival factors (Leifso et al., 2007; Biyani and Madhubala, 2012). If the *ldtyrpip__22_* gene transcription is upregulated in metacyclogenesis as for its ortholog *LmjF.22.0250* in *L. major* (Inbar et al., 2017), it may be involved in a process preparing the parasite for adaptation to the mammalian host microenvironment, part of which is the transformation to the amastigote form. Interestingly, in this study, *Ld*TyrPIP_22 was recruited to the flagellar pocket soon after the stationary axenic promastigote culture was shifted from 25°C to 37°C (**Figure S8**). This recruitment was more dramatic in axenic amastigote-like *L. donovani* forms (**Figures 7A** inset, **7B**, and **S9B**) identified in *L. donovani* culture subjected to temperature and low pH stress conditions for 24–144 h, pointing to a role of this phosphatase in the MP to amastigote transition. In that sense, *Ld*TyrPIP_22 could be involved in the regulation of endo/exocytosis processes, upregulated when parasites are subjected to temperature stress (Hassani et al., 2011) but, also in mechanisms regulating flagellum disassembly with flagellar pocket maintenance through remodeling. The latter is a process that follows the demands of minimizing the amastigote cell’s total surface area as an adaptation in the phagolysosome environment (Wheeler et al., 2016). Moreover, the presence of *Ld*TyrPIP_22 epitopes in the short flagellum tip of amastigote-like parasites (**Figures 7B** and **S9B** small arrowheads) suggests a possible role in the communication of the parasite with the parasitophorous phagosome membrane (Gluenz et al., 2010). All the above stated hypotheses merit further extensive investigation in *Leishmania* cells genetically engineered to overexpress *Ld*TyrPIP_22 or dominant negative mutants of *Ld*TyrPIP_22 or in KO parasites with the *ldtyrpip__22_* gene deleted.

The punctate perinucear redistribution of *Ld*TyrPIP_22, consistently observed at different levels, resembles nuclear pores’ staining pattern. In metazoans, a large number of phosphatases have been shown to localize at the Nuclear envelope and this membrane is attracting attention as a cell’s compartment for the control and transduction of several signaling pathways (Sales Gil et al., 2018). Moreover, PIs are localized inside the nucleus and are shown to play important roles in chromatin remodeling, gene transcription, and RNA processing (Castano et al., 2019). PI kinases, phosphatases and phospholipases have also been shown to localize in the nucleus (Barlow et al., 2010). Moreover, given that *Ld*TyrPIP_22 is a P-Tyr phosphatase as well, its nuclear localization may imply a regulatory role through P-Tyr dephophorylation in nuclear processes, as shown for certain mammalian PTPs. Multiple evidence exists for a turnover of phosphotyrosine at the nuclear envelope of intact metazoan cells (Otto et al., 2001).

An unexpected result of this study was the detection of higher amount of *Ld*TyrPIP_22 protein in total lysates of logarithmically growing promastigotes as compared to promastigotes in stationary phase. The latter constitute the promastigotes’ population enriched in metacyclic forms in which the gene transcript of the *L. major* ortholog of *ldtyrpip__22_* (i.e., the *LmjF.22.0250* gene) was observed to be upregulated 2.6 fold (Dillon et al., 2015) in axenic parasites. However, the metacyclic forms in the stationary phase promastigotes of the *L. donovani* complex *Leishmania* spp. constitute a small pool (below 6%–7%) of the total population (Santarem et al., 2014). Even if this small pool of *L. donovani* metacyclics expressed higher levels of *Ld*TyrPIP_22 this would not affect significantly the levels of this protein in the total population of stationary phase promastigotes, mostly consisting of leptomonad- and nectomonad-like forms (Sunter and Gull, 2017). It remains however an interesting finding that the amount of *Ld*TyrPIP_22 protein detected in the promastigotes’ population enriched in dividing *L. donovani* is ~ 2 fold higher as compared to the non-dividing population, a result that merits further investigation.

Before concluding, it worths mentioning a recent interesting study which showed that *L. mexicana* knock out parasites in the *LmxM.22.0250* gene (i.e., the *ldtyrpip__22_ L. mexicana* ortholog) presented severely attenuated virulence in the infection of primary mouse macrophages *in vitro* (Kraeva et al., 2019). This finding highlights further the importance of our study given that the *Ld*TyrPIP_22 homologs from the pathogenic bacteria *M. tuberculosis* and *L. monocytogenes*, MptpB and LipA, are known virulence factors (Beresford et al., 2009; Kastner et al., 2011). If *Ld*TyrPIP_22 is shown to play a role in *L. donovani* virulence it will gain a value as a candidate drug target, especially because it does not have a homolog in higher eukaryotes (Brenchley et al., 2007; Beresford et al., 2010). Other enzymes of PI metabolism in kinetoplastids have already gained attention as candidate antiparasitic drugs (Cestari, 2020).

To sum up, this work describing the enzymatic substrate specificity and subcellular localization of the *Ld*TyrPIP_22 P-Tyr/PI phosphatase, the first such aDSP to be described in *L. donovani*, forms the basis and will serve as a compass for more detailed studies toward the exploration of this phosphatase’s functional role(s). The results of this study point to an involvement of *Ld*TyrPIP_22 in the regulation of the endocytic/exocytic pathways and in the MP to amastigote differentiation, key processes for the parasite’s fitness to complete its life cycle. The Blast analysis we performed showing that *Ld*TyrPIP_22 is highly conserved amongst several *Leishmania* spp. also highlights the importance of this phosphatase in the parasite’s life cycle. Further follow up studies using *L. donovani* strains with genetic modifications in the *ldtyrpip__22_* gene will enable exploration of the *Ld*TyrPIP_22 putative role(s) in PI signaling in *Leishmania* cells. Although PIs constitute 10% of the total phospholipid content of *Leishmania* promastigotes (Zhang and Beverley, 2010), PI metabolism and signaling in these protozoans remain understudied and PI regulatory molecules identified by bioinformatics analyses (Velasquez et al., 2015) (Brenchley et al., 2007) remain poorly characterized. To conclude, this study emphasizes the importance of *Leishmania* phosphatases classified as aDSPs Lipid-like in the Trypanosomatids’ phosphatome (Brenchley et al., 2007) and as Atypical Lipid phosphatases (Beresford et al., 2010) which, given that they are highly divergent from human homologs or have no human homologs at all, may prove to be valuable candidate drug targets for human leishmaniasis treatment, a goal of strong urgency.

## Data Availability Statement

The datasets presented in this study can be found in online repositories. The names of the repository/repositories and accession number(s) can be found in the article/**Supplementary Material**.

## Ethics Statement

The animal study was reviewed and approved by the Hellenic Pasteur Institute's Institutional Animal Bioethics Committee following the EU Directive 2010/63 and the National Law 2013/56.

## Author Contributions

Conceived and designed the experiments: HB, AP, PR, OT, MK, and IT. Performed the experiments: AP, AK, OT, PK, and AD. Analyzed the data: AP, AK, PR, PK, MK, and HB. Contributed reagents/materials/analysis tools: HB, MK, and IT. Wrote the paper: AP and HB. Edited the paper: MK, IT, OT, and PK. Revised the paper: HB and OT. All authors contributed to the article and approved the submitted version.

## Funding

This work was a Bilateral Research & Technology Collaboration Greece-France 2013 grant (no 1811) funded by the Greek General Secretariat for Research and Technology (http://www.gsrt.gr/central.aspx?sId=119I428I1089I323I488743) (HB and IT); IKYDA, the bilateral research promotion program, concluded between the DAAD with Greece and the Greek State Scholarship Foundation (IKY) (2014-2015) (HB and MK); KRIPIS I & II (2013-2019) Development Grants for Research Institutions, funded by the Greek General Secretariat for Research and Technology (HB), the Hellenic Foundation for Research and Innovation (HFRI) under the HFRI PhD Fellowship grant (Fellowship Number: 606) and the “BIOIMAGING-GR: A Greek Research Infrastructure for Visualizing and Monitoring Fundamental Biological Processes (MIS 5002755)”, funded by the Operational Program “Competitiveness, Entrepreneurship and Innovation” (NSRF 2014-2020), co-financed by Greece and the European Union (European Regional Development Fund). The funders had no role in study design, data collection, and analysis, decision to publish, or preparation of the manuscript.

## Conflict of Interest

The authors declare that the research was conducted in the absence of any commercial or financial relationships that could be construed as a potential conflict of interest.

## Footnotes

1
http://tritrypdb.org/tritrypdb/

2
http://www.ebi.ac.uk/Tools/msa/clustalw2/

3
http://www.cbs.dtu.dk/services/SignalP/

4
http://web.expasy.org/protscale/

5
http://www.cbs.dtu.dk/cgi-bin/webface2.fcgi?jobid=57C41FF500007612F0B128BD&wait=20

6
http://lipid.biocuckoo.org/

7
https://www.graphpad.com/guides/prism/7/curve-fitting/reg_michaelis_menten_enzyme.htm

8
http://icy.bioimageanalysis.org

9
https://www.fpbase.org/protein/mrfp1/

## Supplementary Material

Supplementary Figure 1 Bioinformatics analysis of the *LdT*yrPIP*_*22 active site including the P-loop region HCXXGKDR. **(A)** ClustalW multiple sequence alignment of the *L. donovani* including the P-loop (strain LG13) sequence with homologs from *Mycobacterium tuberculosis* (MptpB, GenBank: CCC62750.1), *Listeria monocytogenes* [lmo1800, NP_465325; lmo1935, NP_465459)], *Yersinia pestis* KIM10+ (WP_002228183), and *Bacillus anthracis* strain Ames (NP_845680). The aa sequences were obtained from the Genbank database and were analyzed/edited using the software BioEdit Sequence Alignment Editor. P-loop signature amino acids are framed by a blue box; **(B)** Rooted phylogenetic tree (UPGMA) from a multiple sequence alignment of the *Ld*TyrPIP_22 orthologs from pathogenic bacteria.

Supplementary Figure 2 Assessment of solubility of the *Ld*TyrPIP_22-His and 15N*-Ld*TyrPIP_22-His recombinant proteins in *E. coli* BL21 lysates. **(A)**The solubility of the r*Ld*TyrPIP_22-His produced in bacteria was tested after 16 h induction with 0.1 mM IPTG at 20 ^o^C, 25 ^o^C and 37 ^o^C. At the end of the induction, bacteria were lysed by sonication, soluble and insoluble fractions were separated by centrifugation (14 000×*g*, 30 min, 4 °C) and the insoluble fraction was resuspended in the same volume of lysis buffer. Equal volumes from total bacteria lysates (lane 1), soluble faction (lane 2) and insoluble fraction (lane 3) were analyzed by SDS-PAGE (12% w/v) followed by WB using the a-*Ld*TyrPIP_22 mouse serum (1:1200). The positions of the MW markers (kDa) are indicated on the left and right of the panels. Two protein species were revealed to be induced, recovered mostly in the insoluble fraction at all 3 temperatures; **(B)** The solubility of 15N-*Ld*TyrPIP_22 produced by bacteria was tested after 16 h induction at 25 °C without IPTG. Equal volumes from total bacteria lysates (lane 1) soluble faction (lane 2) and insoluble fraction (lane 3) were analyzed by SDS-PAGE (12% w/v) followed by WB using the a-His mAb (1.0 *μ*g/mL). The arrow indicates the protein band corresponding to 15N-*Ld*TyrPIP_22 in the part of the blot within the 30-45 kDa range; **(C)** Testing the effect of the length of induction to the solubility of 15N-*Ld*TyrPIP_22 in bacteria lysates. WB of the soluble and pellet fractions from BL21-pTriEx-N15-*ld*t*yrpip__22_*after incubation at 25 °C for 16 h and addition of IPTG (0.5 mM) for the indicated hours. Proteins were analyzed on 12% w/v SDS PAGE and transferred to nitrocellulose which was subsequently incubated with the a-His mAb (1.0 *μ*g/mL). S: soluble supernatant, P: insoluble pellet. Arrow indicates the protein band corresponding to 15N-*Ld*TyrPIP_22.

Supplementary Figure 3
**(A, B)**, Time course of dephosphorylation of four different PIs by the bacterially expressed recombinant human PRL-3 phosphatase (A), or two P-Tyr peptides (1 mM) by the bacterially expressed recombinant human PTP1B (B). The aa sequences for the pTyr2 and pTyr4 peptides are shown in Table S3. The assays were performed as described in **Figure 2**. **(C)**, Effect of pH in the rN15-*Ld*TyrPIP_22-8His phosphatase activity at 37°C. The rN15-*Ld*TyrPIP_22-8His phosphatase activity was assayed in 1 ml reaction volume with *p*NPP as substrate at 37°C (pH 4-8) and at 25°C (pH 6) in parallel as described in *Materials and Methods*. The absorbance of the reaction product (*p*-nitrophenolate = *p*NP) in the supernatant was measured at λ=405 nm. Error bars represent standard deviations calculated from results acquired from four independent experiments performed in duplicates with three different batches of purified recombinant protein.

Supplementary Figure 4 Testing of a-*Ld*TyrPIP_22 Abs’ reactivity **(A)** Western Blot analysis of the a-*Ld*TyrPIP_22 pAbs reactivity (rabbit and mouse polyclonals) in detecting the rN15-*Ld*TyrPIP_22-His protein produced in *E. coli* BL21 cells carrying the pTriEx1.1-rN15-*ldtyrpip__22_*plasmid. Bacteria were grown at 25^o^C for 16 h and induced with 0.5 mM IPTG (4 h, 25^o^C) to express the rN15-*Ld*TyrPIP_22-His protein. Subsequently, the rN15-*Ld*TyrPIP_22-His protein was affinity purified on Ni^2+^ NTA agarose beads from the bacteria lysates (Materials and Methods). Bacteria lysates (lane 1, left panel) or Ni^2+^ NTA beads with rN15-*Ld*TyrPIP_22-His bound on them (lane 2, left panel) were analyzed by 12% SDS-PAGE and transferred on nitrocellulose membranes. Membrane strips with bacteria lysates were probed with the affinity purified rabbit pAb [0.2 & 0,1 *μ*g/ml from the 5^th^ and 1^st^ bleed, (i & ii respectively, middle panel)]. Additionally strips with rN15-*Ld*TyrPIP_22-His (material shown in lane 2 of left panel) were probed with the mouse pAb [dilution 1:100 (iii) & 1:1000 (iv) right panel]. HRP conjugated a-rabbit and a-mouse pAbs were used as secondary Abs. In all the panels the Ab’s reactivity was revealed by the chromogenic DAB method. Arrow heads point to the rN15-*Ld*TyrPIP_22-His band. **(B)** The a-*Ld*TyrPIP_22 pAbs (rabbit and mouse) were used to detect *Ld*TyrPIP_22 epitopes in axenic *L. donovani* static phase promastigotes by IF and confocal microscopy. IF staining of fixed cells permeabilized with 0.1% (v/v) Triton X-100 was performed with the a-*Ld*TyrPIP_22 mouse pAb (1:250) and the a-*Ld*TyrPIP_22 rabbit pAb (1:70) used together. As secondary Abs were used a-mouse Ab conjugated to Alexa Fluor^®^ 633 and an a-rabbit Alexa Fluor 546 respectively. Nuclear and kinetoplast DNA was stained with DAPI. Images were acquired with a TCS SP confocal microscopy by z-scanning performed at 0.3 *μ*m step size. Single optical sections from representative images are shown. Single FL and phase contrast images are presented in Black and White (BW) while merged FL images are shown in color. The 1^st^ Ab used in each case or the dye used for staining are shown at the top in color corresponding to the respective FL signal. Arrow heads point to the flagellar pocket/beginning of flagellum while arrows point to kinetoplast (k) and Nucleus (N). Scale bar size: 8 μm.

Supplementary Figure 5 Localization of *Ld*TyrPIP_22 epitopes in different cell cycle morphological forms of axenic *L. donovani* promastigotes growing at 25^o^C, pH 7. Confocal microscopy images of *L. donovani* promastigotes. IF staining of fixed cells permeabilized with 0.1% (v/v) Triton X-100 was performed with the a-*Ld*TyrPIP_22 mouse (1:250) **(A)** or rabbit (1:70) **(B)** pAbs followed by the corresponding secondary Abs conjugated to Alexa Fluor^®^ 488. Nuclear and kinetoplast DNA was stained with PI. Images were acquired by z-scanning with the TCS-SP confocal microscope at 0.5 *μ*m step size. Single z sections are shown; Details in **B** depict 2X magnifications of the framed areas. Phase contrast and FL images are presented in BW. Merged images of FL or Phase and FL are shown in color. The molecule highlighted in each image or the dye used for staining is indicated at the top in the same color of the respective FL signal. Arrow heads point to the kinetoplast while short arrows point to the flagellar pocket. Long arrows point to the kinetoplast (k) and Nucleus (N). Scale bar size: 8 *μ*m

Supplementary Figure 6 Localization of *Ld*TyrPIP_22 epitopes in promastigotes growing at 25^o^C, pH 7; co staining for *Ld*Tubulin. Confocal microscopy images of axenic *L. donovani* promastigotes from logarithmic **(A)** and stationary phase **(B, C)** cultures growing at 25^o^C and pH 7. IF staining of fixed cells permeabilized with 0.1% (v/v) Triton X-100 was performed with the a-*Ld*TyrPIP_22 rabbit pAb (2 μg/ml) and a-*Ld*Tubulin mouse mAb (1:2000) followed by an a-rabbit Alexa Fluor 546 and an a-mouse Ab conjugated to Alexa Fluor^®^ 488 respectively. Nuclear and kinetoplast DNA was stained with Hoechst 33342 (1:5000). Images were acquired with the Leica TCS SP8 confocal microscope by z-scanning performed at 0.3 μm step size. A single optical section of a procyclic-like promastigote with two flagella is presented in **(A)** while in **(B)** and **(C)** are shown max projections of 7 optical sections from a leptomonad-like **(B)** and a nectomonad-like **(C)** promastigotes. Insets in **(A)**: the framed area with the FL signal intensified so that the two flagella are distinguished. Single FL images are shown in BW and merged in color. The molecule highlighted in each image or the dye used for staining is indicated at the top in the color of the respective FL signal. Arrowheads point to the beginning of flagellum/flagelar pocket region. Arrows point to kinetoplast (k) and Nucleus (N). Scale bar size: 10 μm.

Supplementary Figure 7 Localization of *Ld*TyrPIP_22 in stationary phase *L. donovani* promastigotes stained with the FM4-64FX marker of the endocytic pathway. Confocal microscopy images of axenic *L. donovani* promastigotes grown for 192 h at 25^o^C, (pH 7) and stained alive with the FM4-64FX red fluorescent dye (20 μg/ml, Molecular Probes F34653) for 1 min at 4^o^C **(A)** or 5 min at 25^o^C **(B)**. After removing the dye, promastigotes were washed with ice-cold PBS and fixed with PFA 2% w/v in PBS for 10 min on ice. IF staining of fixed cells permeabilized with 0.1% (v/v) Triton X-100 was performed with the a-*Ld*TyrPIP_22 mouse pAb (1:200) followed by an a-mouse Ab conjugated to Alexa Fluor^®^ 488. The molecule highlighted in each image or the dye used for staining is indicated at the top in the same color as the respective FL signal. Nuclear and kinetoplast DNA was stained with Hoechst 33342 (1:5000). Images were acquired with the Leica TCS SP8 confocal microscope by z-scanning performed at 0.3 μm step size. Single z-sections are shown. Arrowheads point to the beginning of flagellum/flagelar pocket region. Arrows point to kinetoplast (k) and Nucleus (N). Scale bar size: 10 μm.

Supplementary Figure 8 Localization of *Ld*TyrPIP_22 in axenic stationary phase *L. donovani* promastigotes after a quick thermal shift at 37^o^C. Confocal microscopy images of *L. donovani* grown for 192 h at 25^o^C (pH 7) and after a 15 min incubation at 37^o^C (pH 7). IF staining of fixed cells permeabilized with 0.1% (v/v) TritonX-100 was performed with the a-*Ld*TyrPIP_22 mouse pAb (1:200) and a-GAPDH rabbit pAb (1:200) followed by an a-mouse Ab conjugated to Alexa Fluor^®^ 488 and an a-rabbit Alexa Fluor 633 respectively (here presented in red pseudocolor in the merged image). Nuclear and kinetoplast DNA was stained with Hoechst 33342 (1:5000). Single color FL images are presented in BW while merged FL or Phase-FL signals are shown in color. The molecule highlighted in each image or the dye used for staining is indicated at the top in the same color as the respective FL signal. Images were acquired with the Leica TCS SP8 confocal microscope by z-scanning performed at 0.3 μm step size. Single optical sections are shown. Arrowheads point to the beginning of flagellum/flagelar pocket region. Arrows point to kinetoplast (k) and Nucleus (N). Scale bar size: 10 μm.

Supplementary Figure 9 Localization of *Ld*TyrPIP_22 epitopes in axenic amastigote-like forms; co staining for *Ld*Tubulin. Confocal microscopy images of *L. donovani* grown for 5 days at 37^o^C and pH 5.5. IF staining of fixed cells permeabilized with 0.1% (v/v) TritonX-100 was performed as described in Figure S6. The molecule highlighted in each image or the dye used for staining is indicated at the top in the same color as the respective FL signal. Single FL and phase contrast images are presented in BW. Images were acquired with a Leica TCS SP5 confocal microscope by z-scanning performed at 0.3 μm step size. Single z-sections are shown. (**A** a, b): Two different z sections of the same parasite. Arrow heads point to the remaining short flagellum. **(B)** A dividing parasite with amastigote-like morphology. Arrow head points to the tip of the short flagellum. Scale bar size: 5 μm.

## Abbreviations

*Ld*TyrPIP_22, *Leishmania donovani* Tyrosine and Phosphoinositide Phosphatase in chromosome 22; DSP, dual-specificity phosphatase; aDSP, atypical dual-specificity phosphatase; PTPs, Protein Tyrosine Phosphatases; P-Tyr; phosphotyrosine; P-Ser, phosphoserine; P-Thr, phosphotheronine; PI(s), phosphoinositide(s); ALPs, atypical lipid phosphatases; PIPs, PI phosphatases; wt, wild type; ORF, open reading frame; aa, amino acids; Ab, antibody; pAb, polyclonal antibody; mRFP1, monomeric red fluorescent protein; GFP, green fluorescent protein; PFA, paraformaldehyde; ATCC, American Type Culture Collection; MW, Molecular Weight; WB, western blot; h, hours; PI(3)P, phosphatidylinositol 3‐phosphate; PI(4)P, phosphatidylinositol 4‐phosphate; PI(3,5)P_2,_ phosphatidylinositol 3,5‐bisphosphate_;_ IF, immunofluorescence; FL, Fluorescence; BW, black and white; KO, knock out.
