# Supplementary figures and images for: The Leishmania donovani LDBPK_220120.1 Gene Encodes for an Atypical Dual Specificity Lipid-Like Phosphatase Expressed in Promastigotes and Amastigotes; Substrate Specificity, Intracellular Localizations, and Putative Role(s)
Source: Front Cell Infect Microbiol. 2021 Mar 25;11:591868. doi: 10.3389/fcimb.2021.591868 (PMC8027504; doi:10.3389/fcimb.2021.591868)

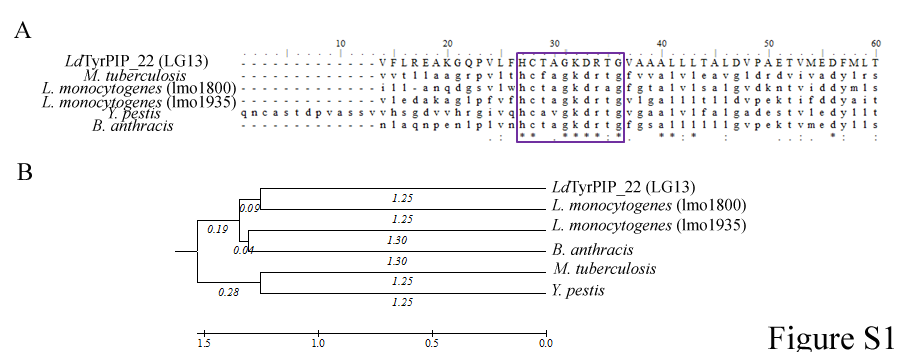

Supplement: Supplementary Figure 1 — Bioinformatics analysis of the LdTyrPIP_22 active site including the P-loop region HCXXGKDR. (A) ClustalW multiple sequence alignment of the L. donovani including the P-loop (strain LG13) sequence with homologs from Mycobacterium tuberculosis (MptpB, GenBank: CCC62750.1), Listeria monocytogenes [lmo1800, NP_465325; lmo1935, NP_465459)], Yersinia pestis KIM10+ (WP_002228183), and Bacillus anthracis strain Ames (NP_845680). The aa sequences were obtained from the Genbank database and were analyzed/edited using the software BioEdit Sequence Alignment Editor. P-loop signature amino acids are framed by a blue box; (B) Rooted phylogenetic tree (UPGMA) from a multiple sequence alignment of the LdTyrPIP_22 orthologs from pathogenic bacteria. [file Image_1.tif]

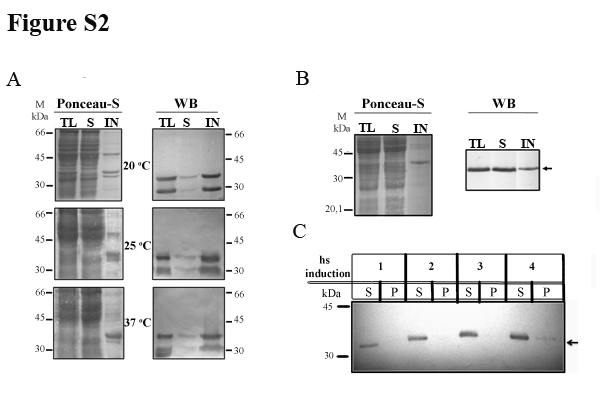

Supplement: Supplementary Figure 2 — Assessment of solubility of the LdTyrPIP_22-His and 15N-LdTyrPIP_22-His recombinant proteins in E. coli BL21 lysates. (A)The solubility of the rLdTyrPIP_22-His produced in bacteria was tested after 16 h induction with 0.1 mM IPTG at 20 oC, 25 oC and 37 oC. At the end of the induction, bacteria were lysed by sonication, soluble and insoluble fractions were separated by centrifugation (14 000×g, 30 min, 4 °C) and the insoluble fraction was resuspended in the same volume of lysis buffer. Equal volumes from total bacteria lysates (lane 1), soluble faction (lane 2) and insoluble fraction (lane 3) were analyzed by SDS-PAGE (12% w/v) followed by WB using the a-LdTyrPIP_22 mouse serum (1:1200). The positions of the MW markers (kDa) are indicated on the left and right of the panels. Two protein species were revealed to be induced, recovered mostly in the insoluble fraction at all 3 temperatures; (B) The solubility of 15N-LdTyrPIP_22 produced by bacteria was tested after 16 h induction at 25 °C without IPTG. Equal volumes from total bacteria lysates (lane 1) soluble faction (lane 2) and insoluble fraction (lane 3) were analyzed by SDS-PAGE (12% w/v) followed by WB using the a-His mAb (1.0 μg/mL). The arrow indicates the protein band corresponding to 15N-LdTyrPIP_22 in the part of the blot within the 30-45 kDa range; (C) Testing the effect of the length of induction to the solubility of 15N-LdTyrPIP_22 in bacteria lysates. WB of the soluble and pellet fractions from BL21-pTriEx-N15-ldtyrpip_22after incubation at 25 °C for 16 h and addition of IPTG (0.5 mM) for the indicated hours. Proteins were analyzed on 12% w/v SDS PAGE and transferred to nitrocellulose which was subsequently incubated with the a-His mAb (1.0 μg/mL). S: soluble supernatant, P: insoluble pellet. Arrow indicates the protein band corresponding to 15N-LdTyrPIP_22. [file Image_2.tif]

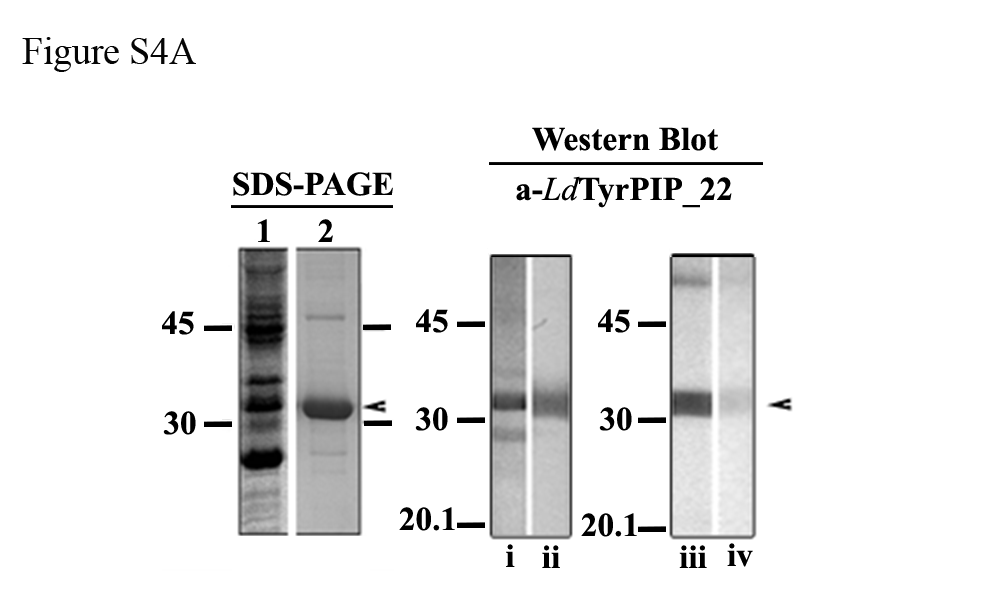

Supplement: Supplementary Figure 3 — (A, B), Time course of dephosphorylation of four different PIs by the bacterially expressed recombinant human PRL-3 phosphatase (A), or two P-Tyr peptides (1 mM) by the bacterially expressed recombinant human PTP1B (B). The aa sequences for the pTyr2 and pTyr4 peptides are shown in Table S3. The assays were performed as described in Figure 2 . (C), Effect of pH in the rN15-LdTyrPIP_22-8His phosphatase activity at 37°C. The rN15-LdTyrPIP_22-8His phosphatase activity was assayed in 1 ml reaction volume with pNPP as substrate at 37°C (pH 4-8) and at 25°C (pH 6) in parallel as described in Materials and Methods. The absorbance of the reaction product (p-nitrophenolate = pNP) in the supernatant was measured at λ=405 nm. Error bars represent standard deviations calculated from results acquired from four independent experiments performed in duplicates with three different batches of purified recombinant protein. [file Image_3.tif]

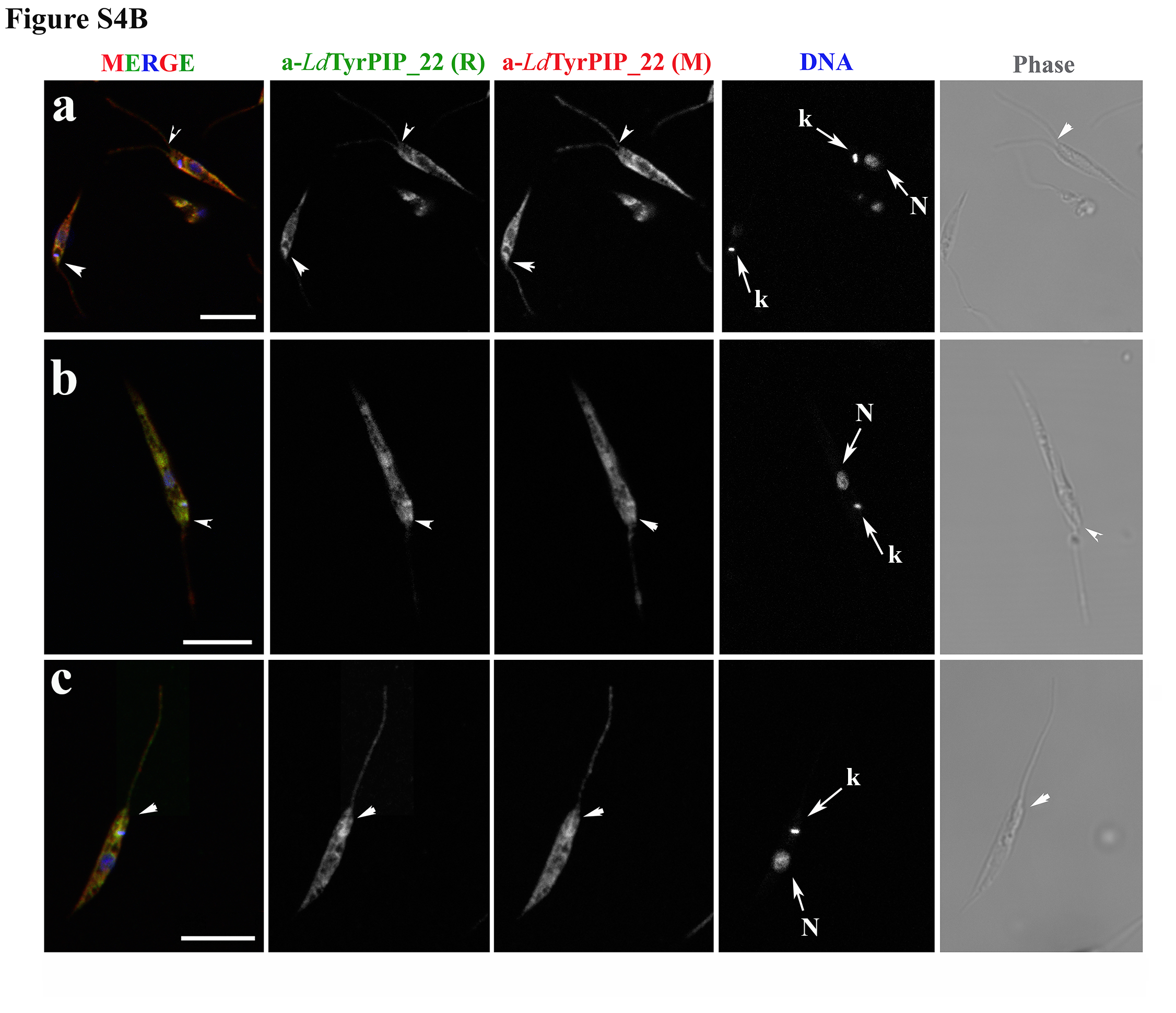

Supplement: Supplementary Figure 4 — Testing of a-LdTyrPIP_22 Abs’ reactivity (A) Western Blot analysis of the a-LdTyrPIP_22 pAbs reactivity (rabbit and mouse polyclonals) in detecting the rN15-LdTyrPIP_22-His protein produced in E. coli BL21 cells carrying the pTriEx1.1-rN15-ldtyrpip_22plasmid. Bacteria were grown at 25oC for 16 h and induced with 0.5 mM IPTG (4 h, 25oC) to express the rN15-LdTyrPIP_22-His protein. Subsequently, the rN15-LdTyrPIP_22-His protein was affinity purified on Ni2+ NTA agarose beads from the bacteria lysates (Materials and Methods). Bacteria lysates (lane 1, left panel) or Ni2+ NTA beads with rN15-LdTyrPIP_22-His bound on them (lane 2, left panel) were analyzed by 12% SDS-PAGE and transferred on nitrocellulose membranes. Membrane strips with bacteria lysates were probed with the affinity purified rabbit pAb [0.2 & 0,1 μg/ml from the 5th and 1st bleed, (i & ii respectively, middle panel)]. Additionally strips with rN15-LdTyrPIP_22-His (material shown in lane 2 of left panel) were probed with the mouse pAb [dilution 1:100 (iii) & 1:1000 (iv) right panel]. HRP conjugated a-rabbit and a-mouse pAbs were used as secondary Abs. In all the panels the Ab’s reactivity was revealed by the chromogenic DAB method. Arrow heads point to the rN15-LdTyrPIP_22-His band. (B) The a-LdTyrPIP_22 pAbs (rabbit and mouse) were used to detect LdTyrPIP_22 epitopes in axenic L. donovani static phase promastigotes by IF and confocal microscopy. IF staining of fixed cells permeabilized with 0.1% (v/v) Triton X-100 was performed with the a-LdTyrPIP_22 mouse pAb (1:250) and the a-LdTyrPIP_22 rabbit pAb (1:70) used together. As secondary Abs were used a-mouse Ab conjugated to Alexa Fluor® 633 and an a-rabbit Alexa Fluor 546 respectively. Nuclear and kinetoplast DNA was stained with DAPI. Images were acquired with a TCS SP confocal microscopy by z-scanning performed at 0.3 μm step size. Single optical sections from representative images are shown. Single FL and phase contrast images are presented in Black and [file Image_4.tif]

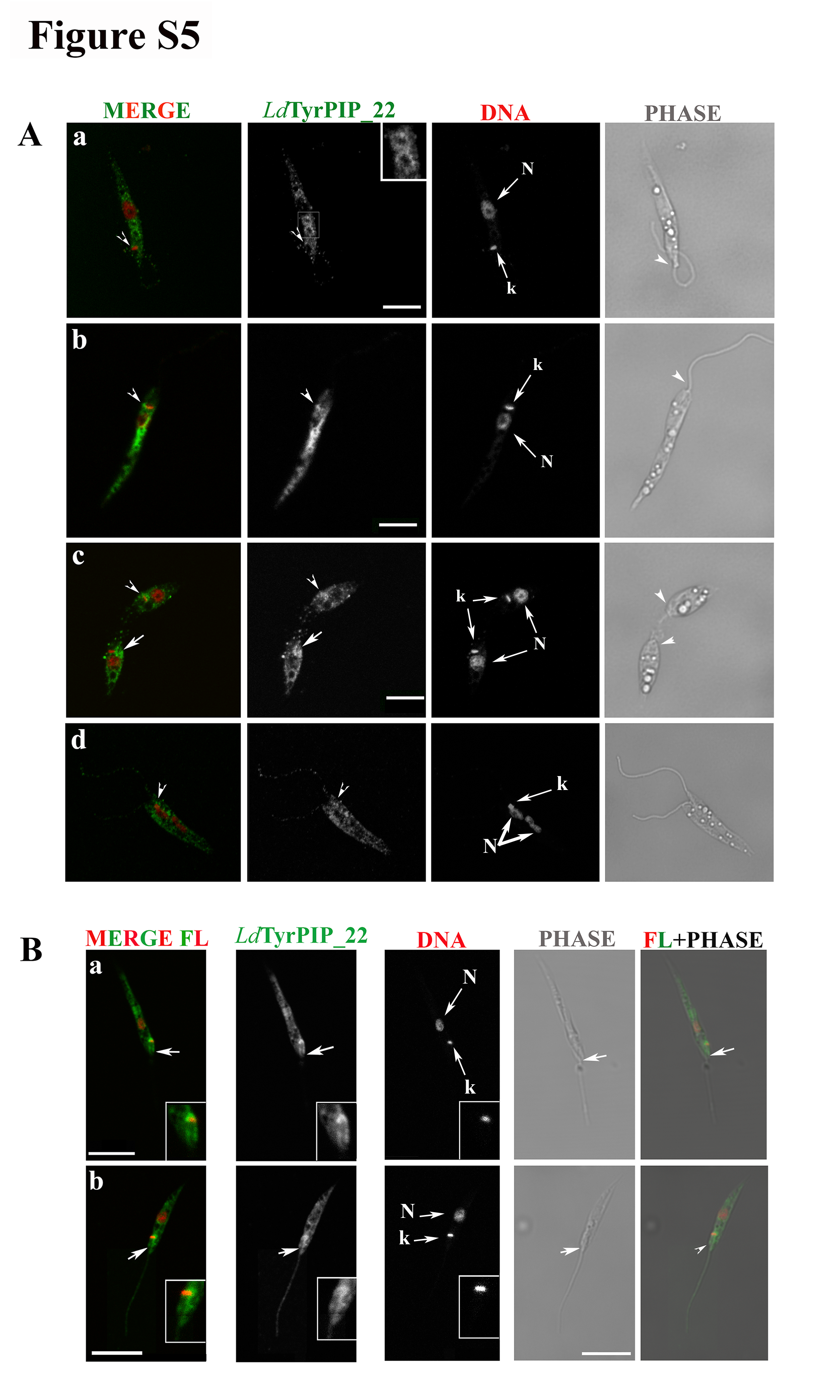

Supplement: Supplementary Figure 5 — Localization of LdTyrPIP_22 epitopes in different cell cycle morphological forms of axenic L. donovani promastigotes growing at 25oC, pH 7. Confocal microscopy images of L. donovani promastigotes. IF staining of fixed cells permeabilized with 0.1% (v/v) Triton X-100 was performed with the a-LdTyrPIP_22 mouse (1:250) (A) or rabbit (1:70) (B) pAbs followed by the corresponding secondary Abs conjugated to Alexa Fluor® 488. Nuclear and kinetoplast DNA was stained with PI. Images were acquired by z-scanning with the TCS-SP confocal microscope at 0.5 μm step size. Single z sections are shown; Details in B depict 2X magnifications of the framed areas. Phase contrast and FL images are presented in BW. Merged images of FL or Phase and FL are shown in color. The molecule highlighted in each image or the dye used for staining is indicated at the top in the same color of the respective FL signal. Arrow heads point to the kinetoplast while short arrows point to the flagellar pocket. Long arrows point to the kinetoplast (k) and Nucleus (N). Scale bar size: 8 μm [file Image_5.tif]

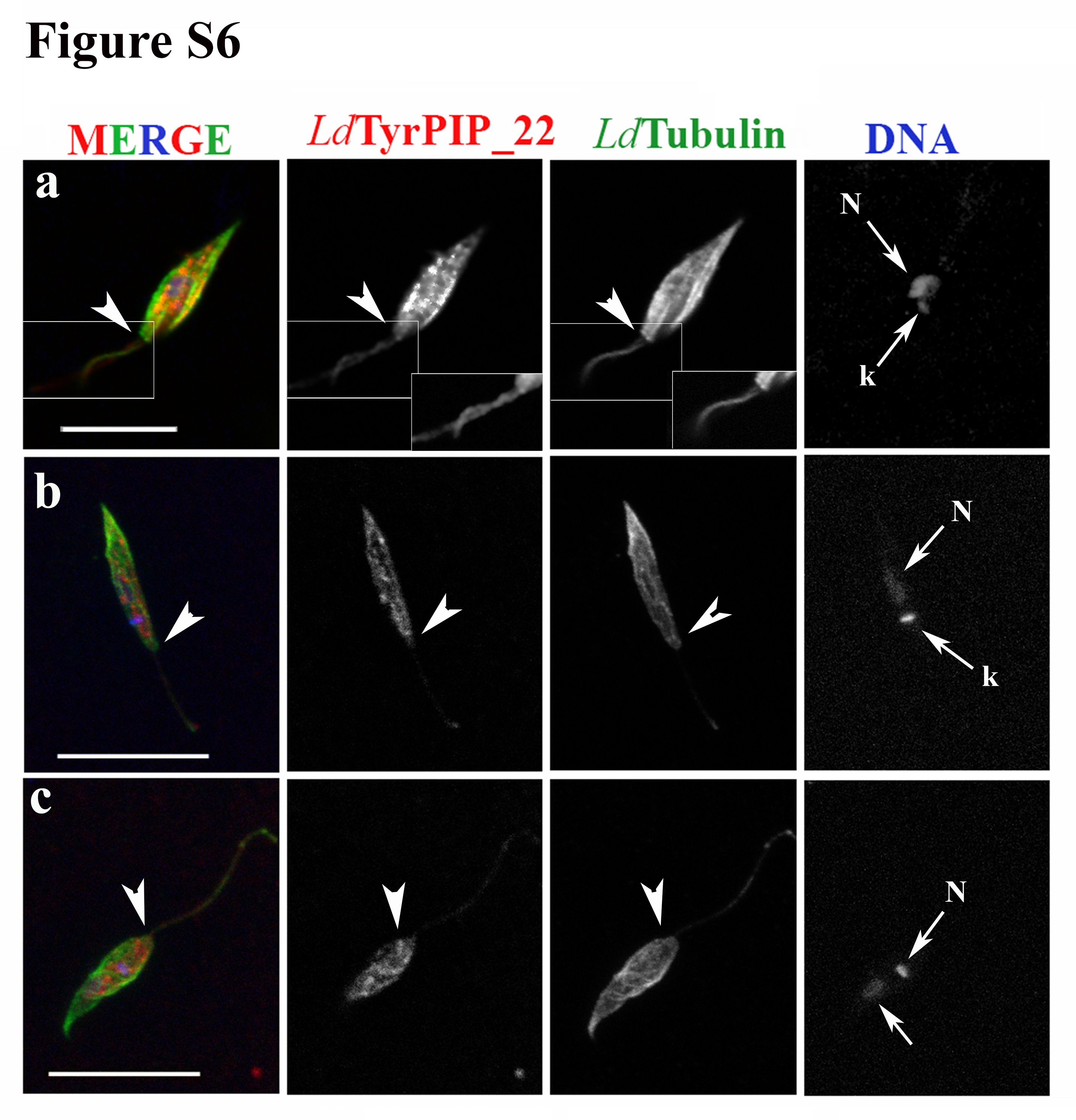

Supplement: Supplementary Figure 6 — Localization of LdTyrPIP_22 epitopes in promastigotes growing at 25oC, pH 7; co staining for LdTubulin. Confocal microscopy images of axenic L. donovani promastigotes from logarithmic (A) and stationary phase (B, C) cultures growing at 25oC and pH 7. IF staining of fixed cells permeabilized with 0.1% (v/v) Triton X-100 was performed with the a-LdTyrPIP_22 rabbit pAb (2 μg/ml) and a-LdTubulin mouse mAb (1:2000) followed by an a-rabbit Alexa Fluor 546 and an a-mouse Ab conjugated to Alexa Fluor® 488 respectively. Nuclear and kinetoplast DNA was stained with Hoechst 33342 (1:5000). Images were acquired with the Leica TCS SP8 confocal microscope by z-scanning performed at 0.3 μm step size. A single optical section of a procyclic-like promastigote with two flagella is presented in (A) while in (B) and (C) are shown max projections of 7 optical sections from a leptomonad-like (B) and a nectomonad-like (C) promastigotes. Insets in (A): the framed area with the FL signal intensified so that the two flagella are distinguished. Single FL images are shown in BW and merged in color. The molecule highlighted in each image or the dye used for staining is indicated at the top in the color of the respective FL signal. Arrowheads point to the beginning of flagellum/flagelar pocket region. Arrows point to kinetoplast (k) and Nucleus (N). Scale bar size: 10 μm. [file Image_6.tif]

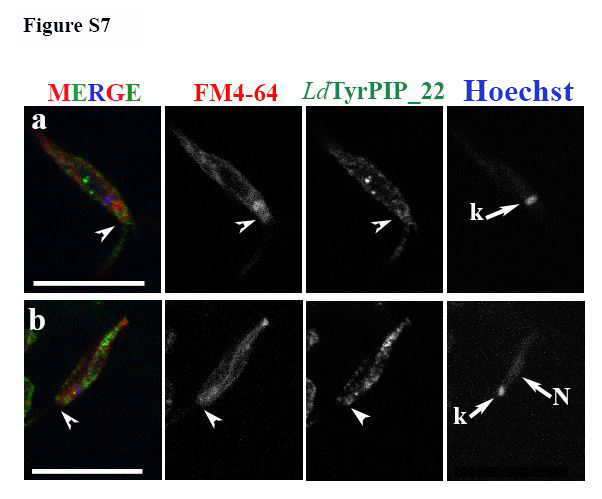

Supplement: Supplementary Figure 7 — Localization of LdTyrPIP_22 in stationary phase L. donovani promastigotes stained with the FM4-64FX marker of the endocytic pathway. Confocal microscopy images of axenic L. donovani promastigotes grown for 192 h at 25oC, (pH 7) and stained alive with the FM4-64FX red fluorescent dye (20 μg/ml, Molecular Probes F34653) for 1 min at 4oC (A) or 5 min at 25oC (B). After removing the dye, promastigotes were washed with ice-cold PBS and fixed with PFA 2% w/v in PBS for 10 min on ice. IF staining of fixed cells permeabilized with 0.1% (v/v) Triton X-100 was performed with the a-LdTyrPIP_22 mouse pAb (1:200) followed by an a-mouse Ab conjugated to Alexa Fluor® 488. The molecule highlighted in each image or the dye used for staining is indicated at the top in the same color as the respective FL signal. Nuclear and kinetoplast DNA was stained with Hoechst 33342 (1:5000). Images were acquired with the Leica TCS SP8 confocal microscope by z-scanning performed at 0.3 μm step size. Single z-sections are shown. Arrowheads point to the beginning of flagellum/flagelar pocket region. Arrows point to kinetoplast (k) and Nucleus (N). Scale bar size: 10 μm. [file Image_7.tif]

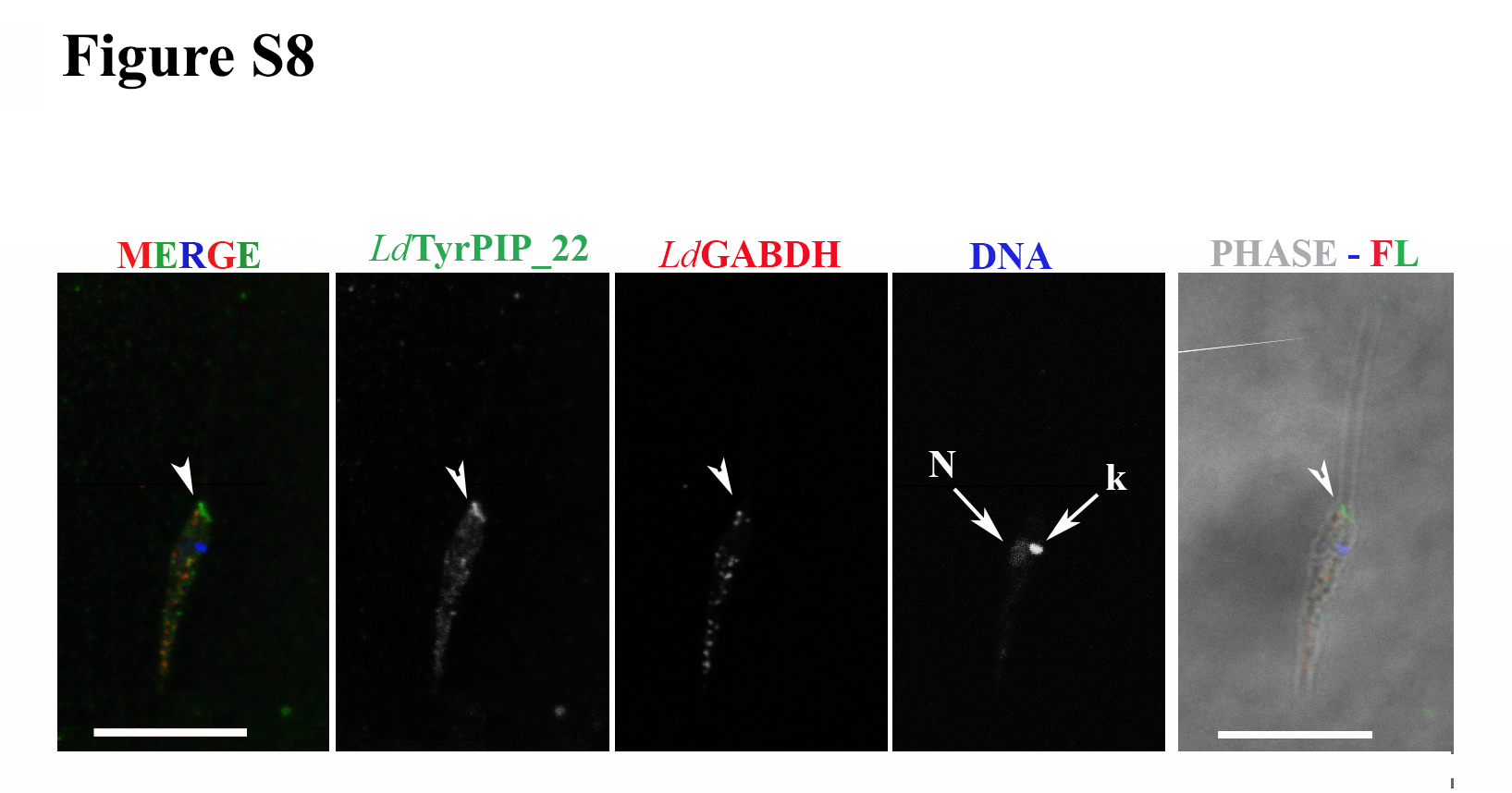

Supplement: Supplementary Figure 8 — Localization of LdTyrPIP_22 in axenic stationary phase L. donovani promastigotes after a quick thermal shift at 37oC. Confocal microscopy images of L. donovani grown for 192 h at 25oC (pH 7) and after a 15 min incubation at 37oC (pH 7). IF staining of fixed cells permeabilized with 0.1% (v/v) TritonX-100 was performed with the a-LdTyrPIP_22 mouse pAb (1:200) and a-GAPDH rabbit pAb (1:200) followed by an a-mouse Ab conjugated to Alexa Fluor® 488 and an a-rabbit Alexa Fluor 633 respectively (here presented in red pseudocolor in the merged image). Nuclear and kinetoplast DNA was stained with Hoechst 33342 (1:5000). Single color FL images are presented in BW while merged FL or Phase-FL signals are shown in color. The molecule highlighted in each image or the dye used for staining is indicated at the top in the same color as the respective FL signal. Images were acquired with the Leica TCS SP8 confocal microscope by z-scanning performed at 0.3 μm step size. Single optical sections are shown. Arrowheads point to the beginning of flagellum/flagelar pocket region. Arrows point to kinetoplast (k) and Nucleus (N). Scale bar size: 10 μm. [file Image_8.tif]

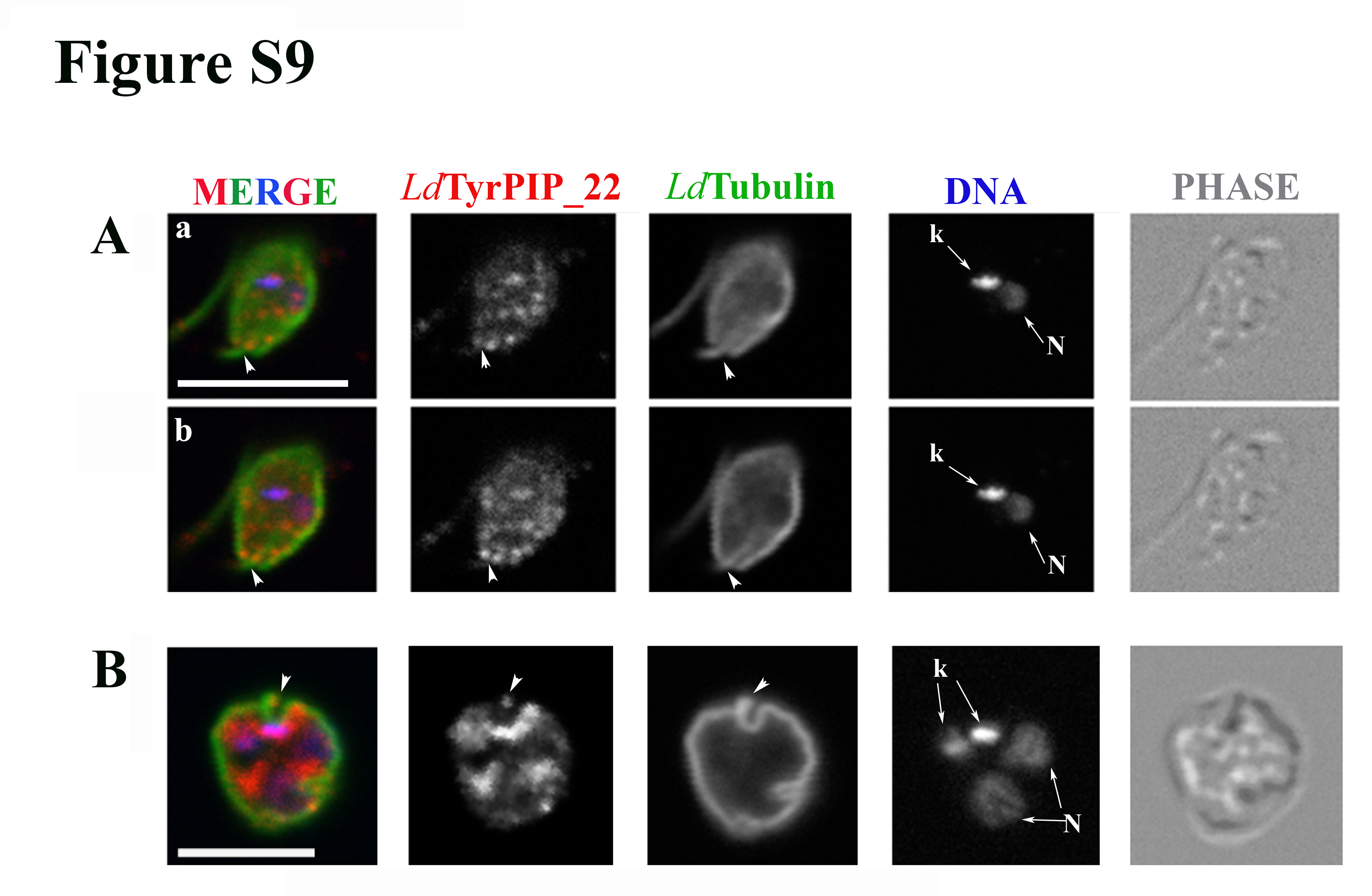

Supplement: Supplementary Figure 9 — Localization of LdTyrPIP_22 epitopes in axenic amastigote-like forms; co staining for LdTubulin. Confocal microscopy images of L. donovani grown for 5 days at 37oC and pH 5.5. IF staining of fixed cells permeabilized with 0.1% (v/v) TritonX-100 was performed as described in Figure S6. The molecule highlighted in each image or the dye used for staining is indicated at the top in the same color as the respective FL signal. Single FL and phase contrast images are presented in BW. Images were acquired with a Leica TCS SP5 confocal microscope by z-scanning performed at 0.3 μm step size. Single z-sections are shown. (A a, b): Two different z sections of the same parasite. Arrow heads point to the remaining short flagellum. (B) A dividing parasite with amastigote-like morphology. Arrow head points to the tip of the short flagellum. Scale bar size: 5 μm. [file Image_9.tif]

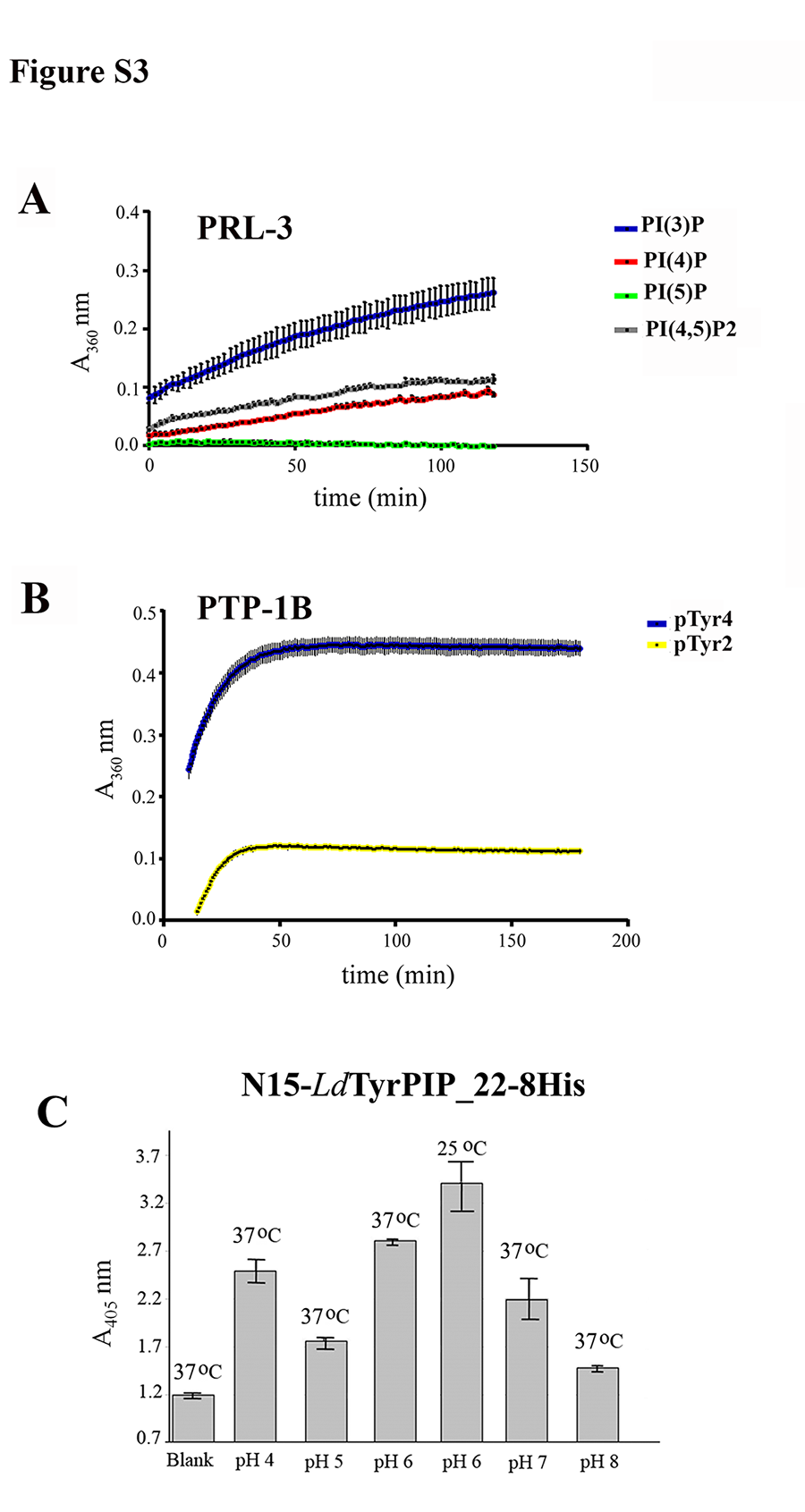

Supplement: Supplementary file 10 [file Image_10.tif]
