# Supplementary material for: The Leishmania donovani LDBPK_220120.1 Gene Encodes for an Atypical Dual Specificity Lipid-Like Phosphatase Expressed in Promastigotes and Amastigotes; Substrate Specificity, Intracellular Localizations, and Putative Role(s)
Source: Front Cell Infect Microbiol. 2021 Mar 25;11:591868. doi: 10.3389/fcimb.2021.591868 (PMC8027504; doi:10.3389/fcimb.2021.591868)
Supplement: Supplementary file 11 [file Table_1.docx]

**SUPLEMENTARY TABLES**

**Table S1**. **Primer sequences used in the PCR reactions**

| **Primer** | | **Nucleic acid sequence** |
| --- | --- | --- |
| For (1) *Ld*TyrPIP_22 (cloning in pTriEX1.1) | | 5’ GAAGATCTCCATGCCCGCCCACAAGCGTATCG 3’ |
| For (2) *Ld*TyrPIP_22 (cloning in pTriEX1.1) | | 5’ GAAGATCTACCATGCCCGCCCACAAGCGTATCG 3’ |
| Rev *Ld*TyrPIP_22 (cloning in pTriEX1.1) | | 5’ CCGCTCGAGTGAGCTCGATGAGGTCG 3’ |
| For *Ld*TyrPIP_22 (cloning in pLexsy-sat-mRFP1) | 5’ GAAGATCTCCATGCCCGCCCACAAGCGTATCG 3’ |  |
| Rev *Ld*TyrPIP_22 (cloning in pLexsy-sat-mRFP1) | 5’ GAAGATCTTCCTGATCCTGAGCTCGATGAGGTCG 3’ |  |
