# Supplementary material for: The Leishmania donovani LDBPK_220120.1 Gene Encodes for an Atypical Dual Specificity Lipid-Like Phosphatase Expressed in Promastigotes and Amastigotes; Substrate Specificity, Intracellular Localizations, and Putative Role(s)
Source: Front Cell Infect Microbiol. 2021 Mar 25;11:591868. doi: 10.3389/fcimb.2021.591868 (PMC8027504; doi:10.3389/fcimb.2021.591868)
Supplement: Supplementary file 12 [file Table_2.docx]

**Table S2. Sequence comparison of *Ld*TyrPIP_22 ortholog gene products in several *Leishmania* spp.**

| ***Leishmania* spp.** | **Strain** | **gene name** | **% identity of gene product** |
| --- | --- | --- | --- |
| *Leishmania donovani* | Nepalese strain BPK282A1 | *LDBPK_220120.1* | 99.87 |
| *Leishmania infantum* | JPCM5 | *LINF_220007400-T1* | 99.61 |
| *Leishmania tropica* | L590 | *LTRL590_220007500* | 95.75 |
| *Leishmania_gerbilli* | LEM452 | *LGELEM452_220007500* | 95.37 |
| *Leishmania aethiopica* | L147 | *LAEL147_000336600* | 95.24 |
| *Leishmania_arabica* | LEM1108 | *LARLEM1108_220007800* | 94.98 |
| *Leishmania_major* | Friedlin | *LmjF.22.0250:mRNA* | 94.59 |
| *Leishmania_turanica* | LEM423 | *LTULEM423_220007600* | 94.08 |
| *Leishmania_mexicana* | MHOM/ GT/2001/U1103 | *LmxM.22.0250* | 93.44 |
| *Leishmania_amazonensis* | MHOM/BR/71973/M2269 | *LAMA_000419900* | 93.30 |
| *Leishmania_tarentolae* | Parrot-TarII | *LtaP22.0250.mRNA* | 83.91 |
| *Leishmania_enriettii* | LEM3045 | *LENLEM3045_220007500* | 81.34 |
