# Supplementary material for: The Leishmania donovani LDBPK_220120.1 Gene Encodes for an Atypical Dual Specificity Lipid-Like Phosphatase Expressed in Promastigotes and Amastigotes; Substrate Specificity, Intracellular Localizations, and Putative Role(s)
Source: Front Cell Infect Microbiol. 2021 Mar 25;11:591868. doi: 10.3389/fcimb.2021.591868 (PMC8027504; doi:10.3389/fcimb.2021.591868)
Supplement: Supplementary file 13 [file Table_3.docx]

**SUPLEMENTARY TABLES**

**Table S3. Phosphorylated substrates used in the study**

| **Peptide** (as in Figure 2) | **Peptide sequence** | **protein** |
| --- | --- | --- |
| pTyr1 | LGSRA**pY**PHFCA | NOS3 |
| pTyr2 | IEDP**pYpY**GNDSD | LMW-PTP |
| pTyr3 | DPSDN**pY**AEPID | ARHGAP5 |
| pTyr4 | DpY**pY**R | IRK |
| pSer/Thr1 | Ac-IQAAA**pSpT**P | GSK3β |
| pSer/Thr2 | Ac-I**pS**PPPTANL | FAK1 |
| pSer/Thr3 | Biotin-QGRDKYK**pT**LRQIRQG | EZRIN |
